# Synthesis and Evaluation of Thymol-Based Synthetic Derivatives as Dual-Action Inhibitors against Different Strains of *H. pylori* and AGS Cell Line

**DOI:** 10.3390/molecules26071829

**Published:** 2021-03-24

**Authors:** Francesca Sisto, Simone Carradori, Paolo Guglielmi, Mattia Spano, Daniela Secci, Arianna Granese, Anatoly P. Sobolev, Rossella Grande, Cristina Campestre, Maria Carmela Di Marcantonio, Gabriella Mincione

**Affiliations:** 1 Department of Biomedical, Surgical and Dental Sciences, University of Milan, 20122 Milan, Italy; francesca.sisto@unimi.it; 2 Department of Pharmacy, “G. d’Annunzio” University of Chieti-Pescara, Via dei Vestini 31, 66100 Chieti, Italy; rossella.grande@unich.it (R.G.); cristina.campestre@unich.it (C.C.); 3 Department of Chemistry and Technology of Drugs, Sapienza University of Rome, P.le A. Moro 5, 00185 Rome, Italy; paolo.guglielmi@uniroma1.it (P.G.); mattia.spano@uniroma1.it (M.S.); daniela.secci@uniroma1.it (D.S.); arianna.granese@uniroma1.it (A.G.); 4 Institute for Biological Systems, “Annalaura Segre” Magnetic Resonance Laboratory, CNR, 00015 Rome, Italy; anatoly.sobolev@cnr.it; 5 Department of Innovative Technologies in Medicine and Dentistry, “G. d’Annunzio” University of Chieti-Pescara, 66100 Chieti, Italy; dimarcantonio@unich.it (M.C.D.M.); gabriella.mincione@unich.it (G.M.)

**Keywords:** thymol, *Helicobacter pylori*, AGS cells, semi-synthesis, drug resistance, dual-action agents, antimicrobial activity

## Abstract

Following a similar approach on carvacrol-based derivatives, we investigated the synthesis and the microbiological screening against eight strains of *H. pylori*, and the cytotoxic activity against human gastric adenocarcinoma (AGS) cells of a new series of ether compounds based on the structure of thymol. Structural analysis comprehended elemental analysis and ^1^H/^13^C/^19^F NMR spectra. The analysis of structure–activity relationships within this molecular library of 38 structurally-related compounds reported that some chemical modifications of the OH group of thymol led to broad-spectrum growth inhibition on all isolates. Preferred substitutions were benzyl groups compared to alkyl chains, and the specific presence of functional groups at *para* position of the benzyl moiety such as 4-CN and 4-Ph endowed the most anti-*H. pylori* activity toward all the strains with minimum inhibitory concentration (MIC) values up to 4 µg/mL. Poly-substitution on the benzyl ring was not essential. Moreover, several compounds characterized by the lowest minimum inhibitory concentration/minimum bactericidal concentration (MIC/MBC) values against *H. pylori* were also tested in order to verify a cytotoxic effect against AGS cells with respect to 5-fluorouracil and carvacrol. Three derivatives can be considered as new lead compounds alternative to current therapy to manage *H. pylori* infection, preventing the occurrence of severe gastric diseases. The present work confirms the possibility to use natural compounds as templates for the medicinal semi-synthesis.

## 1. Introduction

Thymol, a major natural monoterpene phenol in the essential oils of Lamiaceae and Apiaceae families, has been approved by European Union and Food and Drug Administration as a safe food additive as well as in cosmetics and agriculture industry. Among the terpenes, it has been demonstrated to be clinically relevant as an antimicrobial, antioxidant, and anti-inflammatory agent [1]. As well as its regioisomer carvacrol, thymol could exert an inhibitory effect on Gram-positive and Gram-negative bacteria, but only few studies were oriented toward *H. pylori* [2,3], preferring the use of standardized thymol-containing plant extracts for the eradication. This Gram-negative and microaerophilic pathogen colonizes and survives in the human gastric epithelium, which represents its favourite ecological niche. About 50% of the global population is colonized by the microorganism although in most cases *H. pylori* causes no symptoms [4]. The survival of the microorganism as well as the recalcitrance of the infection are due to *H. pylori* aptitude to adapt itself to the host and to develop resistance towards the antimicrobials commonly used in therapy [5]. The resistance rate to the clinically approved drugs is increasing worldwide and most of the therapeutic failures are ascribed to adaptive mechanisms linked to the biofilm formation [6], which limit the efficacy of the current therapy. The triple therapy based on the administration on proto-pump inhibitor and two antibiotics has been considered the standard therapy for many years, however, the increasing rate of triple-therapy failure was registered in many countries as well as the spreading of *H. pylori* antibiotic resistance. In this context, novel compounds such as components of essential oils endowed with alternative mechanisms of action and the ability to disaggregate lipidic multilayer structures are strongly suggested [7]. More in detail, thymol derivatives have been proposed as inhibitors of CagA and VacA (cytotoxin-associated gene A and vacuolization cytotoxin) oncoproteins of *Helicobacter pylori* [8] and, recently, Bkhaitan et al. also prepared a thymol-metronidazole ester hybrid and assessed its discrete anti-*Helicobacter pylori* activity and limited cytotoxicity on normal cell lines [9].

In addition, *H. pylori* is considered as a class I carcinogen by the World Health Organization and can secrete virulence factors, which not only affect host signalling pathways but can also induce a sustained gastric inflammatory process ultimately leading to the development of cancerous conditions in the stomach and duodenum [10]. In the last years, thymol has been the focus of some research studies on the potential suppression of cancer cell growth [11], ascribing to this molecule the capability to induce apoptosis; cause morphological changes; generate reactive oxygen species; depolarize the potential of mitochondrial membrane; and activate Bax, caspases and PARP (Poly (ADP-ribose) polymerase). It was also tested against the human gastric carcinoma cells (AGS) with contrasting biological results. Indeed, Kang et al. reported an IC_50_ of 400 µM after 24 h of treatment [12], whereas Günes-Bayir et al. disclosed an IC_50_ of 75.63 µM [13]. Despite the reported values, it was evident that the cytotoxic effects on AGS cells were mediated in a concentration-dependent manner by the activation of apoptotic pathways prolonging the sub-G1 phase, generation of reactive oxygen species (ROS) especially at higher doses, and glutathione (GSH) depletion [14].

Starting from these important premises, we aimed at modifying the structure of this well-known natural product in order to achieve a small library of novel dual-action ether compounds, which can address multifaceted pathological conditions such as *H. pylori*-induced gastric cancer and provide a better resistance to the acidic gastric environment.

## 2. Results and Discussion

### 2.1. Chemistry

**Figure 1:**
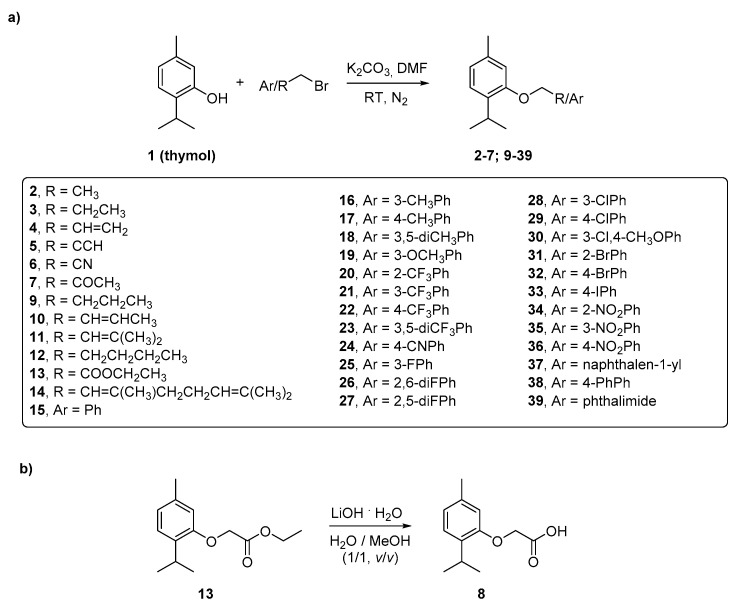
Synthesis of the reported thymol-based compounds **2**–**39**. (**a**) Functionalization of the OH moiety of thymol (**1**); (**b**) ester hydrolysis to provide compound **8**.

For the synthesis of compounds **2**–**39,** we employed the synthetic approach reported in Figure 1. Compounds **2**–**7** and **9**–**39** (Figure 1a) have been synthesized taking advantage of the Williamson etherification procedure previously employed by our group for the synthesis of carvacrol derivatives [15]. Thymol (**1**) was reacted with the proper bromide in dry *N*,*N*’-dimethylformamide (DMF), in the presence of anhydrous potassium carbonate (K_2_CO_3_) and under nitrogen (N_2_) atmosphere. These reactions were performed at room temperature (RT) in order to avoid the reagent degradation. For the synthesis of compound **8** (Figure 1b) we started from compound **13** that underwent the hydrolysis of the ethyl ester group to afford the carboxylic acid moiety. The hydrolysis was performed in mild conditions using lithium hydroxide (LiOH) monohydrate, in a mixture of water and methanol (in the ratio 50:50*, v:v*) at room temperature.

The choice of such substituent was driven by the observation of a greater inhibition of microbial growth after the introduction of bulkier alkyl/benzyl groups on the hydroxyl moiety. The obtained compounds were stable at room temperature. The structures were confirmed by spectral studies (^1^H, ^13^C, and ^19^F NMR), whereas the purity of these compounds was confirmed by combustion analysis, Thin Layer Chromatography (TLC) parameters and, for solid compounds, melting point.

### 2.2. Anti-Helicobacter pylori Activity and Structure-Activity Relationship Studies

After the procedures of purification and characterization and the in silico analysis of drug-likeness, all the compounds (**1**–**39**) were tested in vitro against *H. pylori* growth and the 12 most promising ones also against AGS cells to suggest dual-action agents useful for the treatment of *H. pylori*-related gastric cancer development.

The phenolic terpene thymol and its 38 semi-synthetic derivatives were firstly assayed against eight strains of *H. pylori* (one reference and seven clinical isolates). All the strains were classified according to their antimicrobial susceptibility to metronidazole, clarithromycin, and amoxicillin following the international EUCAST (European Committee on Antimicrobial Susceptibility Testing) breakpoints. Thymol, with respect to carvacrol, displayed a weaker inhibitory activity against all the strains used with MIC and MBC values ranging from 64–128 µg/mL. These results are in accordance with those reported in the literature [16,17,18].

Four clinical isolates, namely F1, F40/499, F4, and F34/497, were the most sensitive to the treatment with thymol (**1**) (MIC/MBC of 64 µg/mL), slightly better than the values reported for other clinical isolates [19]. Similar results were obtained for carvacrol. The derivatives, functionalized at the OH moiety of the parent compound, can be clustered into two groups (alkyloxy and benzyloxy/heteroarylmethyloxy compounds) in order to extrapolate robust structure–activity relationship (SAR) within this scaffold (Table 1). The most important information is that the OH group, as in carvacrol-based derivatives, did not seem to be essential at least to display anti-*H. pylori* activity. Other ether groups can be tolerated suggesting a specific mechanism of action with respect to the direct alteration of the bacterial membrane.

Among the first cluster of alkyloxy derivatives (**2**–**14**), the MIC results demonstrated no or weak efficacy against all the strains as well as their parent compound, despite the presence of branched alkyl chains, unsaturations or specific functional groups (ketone, carboxylic acid, ethyl ester). Only three derivatives (**3**, **9**, **12**) with linear and increasing alkyl chains (Et, *n*-Pr, *n*-Bu) presented improved MIC values with respect to thymol against some clinical isolates (MIC/MBC = 16 µg/mL). Similar results were obtained for the aliphatic derivatives of carvacrol in our previous paper [15].

Among the second cluster of benzyloxy and heteroarylmethyloxy derivatives (**15**–**39**), the simplest and unsubstituted compound (**15**) was slightly more potent than thymol, but the introduction of electron-withdrawing or electron-donating substituents at different positions induced an opposite trend on the inhibitory activity. Substitutions at the *ortho* position of the benzyl group were usually detrimental (compounds **16**, **20**, **31**) except for the potent activity of compound **34** (*o*-NO_2_) on specific strains (MIC range 4–8 µg/mL, MBC range 8–16 µg/mL). Methoxy (**19**), chloro (**28**), and nitro (**35**) at *meta* position did not alter the anti-*Helicobacter pylori* activity, whereas *m*-CF_3_ (**21**) dramatically reduced it and *m*-F (**25**) elicited it. As reported for carvacrol-based compounds, derivatives with *para* substitution were generally more potent (**17**, **29**, **32**), except for **33** and **36**. Two derivatives (**24** and **38**), with 4-CN and 4-Ph respectively, were endowed with the most anti-*H. pylori* activity toward all the considered strains with MIC values up to 4 µg/mL. Poly-substitution on the benzyl ring was not important (**18** and **23**), albeit three compounds (**26**, **27**, **30**) slightly improved the antimicrobial activity with respect to thymol. Finally, the insertion on the OH group of thymol of a naphthalene ring (**37**) was detrimental, whereas the presence of a phthalimide nucleus (**39**) enhanced the activity. Collectively, these data suggested for all the derivatives a bactericidal mechanism of action, considering the MBC/MIC ratio to be between 1–2. This biological activity did not seem to be strictly correlated to the presence of a slightly acidic and polar phenolic OH.

### 2.3. Effects of Thymol and Its Semi-Synthetic Derivatives on AGS Cell Viability

To better assess the dual-action behaviour of these compounds, the selection of the candidates for biological assays was guided by the anti-*Helicobacter pylori* activity (Table 1, compounds highlighted in grey). AGS cells were incubated for 24 h with the specified molecules or with 0.1% dimethyl sulfoxide (DMSO) vehicle (control). Data shown are the means ± SD of three experiments with quintuplicate determinations (Table 2). Thymol (**1**) showed discrete cytotoxic effects by reducing cell viability of AGS cells (IC_50_ = 200 ± 6.5 μM) in a dose-dependent manner, which is in accordance with literature data [12]. It displayed a stronger cytotoxic effect at 24 h with respect to its positional isomer carvacrol (IC_50_ = 300 ± 6.4 μM). Out of 11 thymol derivatives, only three (**9**, **15**, **38**) presented a dose-dependent inhibitory effect on cell viability inferior to thymol. All of them possessed an IC_50_ value higher than the reference drug 5-fluorouracil (5-FU, IC_50_ = 82.3 ± 5.6 μM).

Collectively, the functionalization of the OH moiety with an unsubstituted benzyloxy group (**15**) or a linear alkyloxy chain (**9**) was preferred in eliciting the inhibitory activity below 100 μM. The substitution of the benzyl ring with NO_2_, F, and CN led to a reduction of the biological effect as well as the poly-substitution (**26** and **30**). The change of the benzyl group with a phthalimide (**39**) was detrimental. Interestingly, compound **38** with a 4-Ph on the benzyl ring exerted a comparable activity with respect to the parent compound.

### 2.4. Pan Assay Interference Compounds (PAINS) and Drug-Likeness Evaluation

In addition, we selected three representative and dual-action compounds (**9**, **15,** and **38**) for a broader analysis (SwissADME) of their chemical-physical characteristics and Medicinal Chemistry properties (Table 3) [20,21].

All designed inhibitors have been analyzed by means of theoretical tools regarding the possibility to act as interfering/covalent compounds in different in vitro assays. Indeed, the analysis of substructures to elicit promiscuous pharmacological behaviour, commonly named PAINS, should be mandatory in Medicinal Chemistry for a further and proper development of such compounds in the clinical settings. Our top-rated thymol derivatives were devoid of this property. Moreover, their drug-likeness was evaluated based on the Lipinski’s rule of five, largely met by all the derivatives (for compounds **15** and **38**, MLOGP > 4.15, which approximatively corresponds to CLogP ˃ 5, was the only violation). Log P, calculated by Moriguchi-based computer program (MLOGP), is one of the parameters that can estimate the proper absorption or permeation of a drug candidate [22]. In fact, compound **38** has also a low gastro-intestinal absorption owing to its very high MLOGP value. These features, if focused on drugs acting after oral administration specifically on the *H. pylori*-colonized gastric mucosa, should not be considered negatively. Interestingly, as proposed by the SwissADME tool, these compounds do not display any kind of interaction with permeability glycoprotein (P-gp) that promotes the efflux of cytotoxic drugs out of the cells. This characteristic is suitable to enhance the efficacy of anti-proliferative agents, due to the involvement of this protein in the multi-drug resistance [23]. The other parameters, as reported in the footnote of Table 3, were used for the construction of both the boiled-egg graph and the bioavailability radar. The former allows an easy understanding and visualization of the passive absorption at the GI tract (white area) and the ability to permeate the blood brain barrier (BBB, yellow area). Only compounds **9** and **15** are able to be easily absorbed by the GI and provide the possibility to cross the BBB. Finally, the information obtained from the bioavailability radar graphical output suggests the drug-likeness depiction of the selected compounds including the optimal range of each physical-chemical property (flexibility, size, lipophilicity, polarity, saturation, solubility) essential to provide oral bioavailability. Collectively, these computed data are of interest for the clinical development of compounds acting as antibacterial or anti-cancer.

## 3. Materials and Methods

### 3.1. Chemistry

All reactions were carried out under a positive pressure of nitrogen in washed and oven-dried glassware. All the solvents and high-purity reagents were directly used as supplied by Sigma-Aldrich (Milan, Italy) without further purification. Where mixtures of solvents are specified, the stated ratios are volume:volume. All melting points were measured on a Stuart^®^ melting point apparatus SMP1 and are uncorrected (temperatures are reported in °C). Structural analysis consisted of elemental analysis and ^1^H/^13^C/^19^F NMR spectra. ^1^H and ^13^C-NMR spectra were recorded both at 300 MHz and 75 MHz (Varian Mercury spectrometer, Varian, Santa Clara, CA, USA), while other compounds were analysed at 400 MHz and 101 MHz on a Bruker spectrometer (Milan, Italy), using CDCl_3_ and DMSO-*d_6_*, as the solvents at room temperature. Conversely, ^19^F spectra were recorded on a Bruker AVANCE 600 spectrometer at 564.7 MHz, using CDCl_3_ as the solvent. All the compounds were studied at the final concentration of ~25 mg/mL. ^1^H and ^13^C chemical shifts are expressed as *δ* units (parts per millions) and referenced to the residual solvent signal (CDCl_3_ *δ*_H_ 7.26 ppm and *δ*_C_ 77.2 ppm, DMSO-*d*_6_ *δ*_H_ 3.33 and *δ*_C_ 39.5 ppm), whereas ^19^F chemical shifts are expressed as *δ* units relative to an external standard (CF_3_COOH, *δ−*76.55 ppm). ^1^H spectra are described as follows: *δ*_H_ (spectrometer frequency, solvent): chemical shift/ppm (multiplicity, *J*-coupling constant(s) in Hertz (Hz), number of protons, assignment). ^13^C spectra are described as follows: *δ*_C_ (spectrometer frequency, solvent): chemical shift/ppm (assignment) and are fully proton decoupled. ^19^F spectra are described as follows: *δ*_F_ (spectrometer frequency, solvent): chemical shift/ppm (multiplicity, *J* coupling constant(s) in Hertz, number of fluorine, assignment) and are reported as Supplementary Materials. Multiplets are abbreviated as follows: br—broad; s—singlet; d—doublet; t—triplet; —quartet; td—triplet of doublets; m—multiplet. The exchangeable protons (OH) were assessed by the addition of deuterium oxide. The processing and analyses of the NMR data were carried out with MestreNova. Elemental analyses for C, H, and N were recorded on a Perkin-Elmer 240 B microanalyzer obtaining analytical results within ± 0.5% of the theoretical values for the final compounds. Preparative chromatography was carried out employing silica gel (high purity grade, pore size 60 Å, 230–400 mesh particle size). All the purifications and reactions were carried out by thin layer chromatography (TLC) performed on 0.2-mm-thick silica gel-aluminium backed plates (60 F254). Spot visualization was performed under short and long wavelengths (254 and 365 nm, respectively) ultra-violet irradiation. Where given, systematic compound names were generated by ChemBioDraw Ultra 14.0 following IUPAC conventions.

### 3.2. Synthesis of Thymol Derivatives

#### 3.2.1. General Procedure for the Synthesis of Compounds **2**–**7** and **9**–**39**

To a stirring solution of thymol (**1**, 1.0 equiv.) in dry DMF (10 mL) was added freshly ground and anhydrous potassium carbonate (K_2_CO_3_, 1.1 equiv.) under N_2_ atmosphere. The suspension was stirred for 15 min at room temperature; then, the proper (un)substituted benzyl or alkyl bromide (1.5 equiv.) was added and the reaction stirred until disappearance of the starting reagents, as detected by TLC (24–72 h). Once the reaction was completed, the mixture was poured into ice-cold water (100 mL) and extracted with dichloromethane (DCM, 3 × 20 mL). The organics were collected, dried over sodium sulphate (Na_2_SO_4_), and evaporated in vacuo to afford the crude mixture that was purified through silica gel column chromatography, using proper mixtures of *n*-hexane/ethyl acetate as mobile phase.

#### 3.2.2. Synthesis of Compound **8**

To a stirring solution of ethyl 2-(2-isopropyl-5-methylphenoxy)acetate (**13**, 1.0 equiv.) in 10 mL of methanol was added dropwise a solution of lithium hydroxide (1.2 equiv.) in 10 mL of water. The reaction was stirred at room temperature for 24 h; then, the mixture was concentrated in vacuo to remove methanol and quenched with 3N HCl (15 mL). The precipitate was collected by filtration and washed with *n*-hexane to give the title compound **8**, without further purification requirements.

### 3.3. Characterization Data for Thymol Derivatives ***2***–***39***

*2-ethoxy-1-isopropyl-4-methylbenzene* (**2**): colourless oil, 75% yield. ^1^H-NMR (400 MHz, CDCl_3_): *δ* 1.26–1.28 (m, 6H, 2 × CH_3_), 1.46–1.50 (m, 3H, CH_3_), 2.38 (s, 3H, ArCH_3_), 3.31–3.41 (m, 1H, CH), 4.06–4.11 (m, 2H, OCH_2_), 6.72 (s, 1H, Ar), 6.79 (d, *J* = 8.0 Hz, 1H, Ar), 7.15 (d, *J* = 8.0 Hz, 1H, Ar). ^13^C-NMR (101 MHz, CDCl_3_): *δ* 15.0 (CH_3_), 15.9 (ArCH_3_), 24.1 (2 × CH_3_), 34.2 (CH), 63.5 (OCH_2_), 109.6 (Ar), 117.9 (Ar), 124.1 (Ar), 130.4 (Ar), 147.8 (Ar), 157.1 (Ar). Anal. Calcd for C_12_H_18_O: C, 80.85; H, 10.18. Found: C, 80.79; H, 10.13.

*1-isopropyl-4-methyl-2-propoxybenzene* (**3**): colourless oil, 72% yield. ^1^H-NMR (300 MHz, CDCl_3_): *δ* 1.38–1.43 (m, 3H, CH_3_), 1.58 (d, *J* = 7.2 Hz, 6H, 2 × CH_3_), 2.10–2.19 (m, 2H, CH_2_), 2.66 (s, 3H, ArCH_3_), 3.64–3.71 (m, 1H, CH), 4.21–4.25 (m, 2H, OCH_2_), 6.99 (s, 1H, Ar), 7.06 (d, *J* = 8.1 Hz, 1H, Ar), 7.43 (d, *J* = 7.5 Hz, 1H, Ar). ^13^C-NMR (75 MHz, CDCl_3_): *δ* 11.1 (CH_3_), 21.6 (ArCH_3_), 23.1 (CH_2_), 23.2 (2 × CH_3_), 27.0 (CH), 69.6 (OCH_2_), 112.3 (Ar), 121.2 (Ar), 126.1 (Ar), 134.3 (Ar), 136.4 (Ar), 156.5 (Ar). Anal. Calcd for C_13_H_20_O: C, 81.20; H, 10.48. Found: C, 81.20; H, 10.45.

*2-(allyloxy)-1-isopropyl-4-methylbenzene* (**4**): colourless oil, 64% yield. ^1^H-NMR (300 MHz, CDCl_3_): *δ* 1.54 (d, *J* = 6.6 Hz, 6H, 2 × CH_3_), 2.61 (s, 3H, ArCH_3_), 3.62–3.72 (m, 1H, CH), 4.77–4.80 (m, 2H, OCH_2_), 5.52–5.56 (m, 1H, =CH_2_), 5.70–5.77 (m, 1H, =CH_2_), 6.30–6.41 (m, 1H, =CH), 6.94 (s, 1H, Ar), 7.04 (d, *J* = 7.8 Hz, 1H, Ar), 7.40 (d, *J* = 8.1 Hz, 1H, Ar). ^13^C-NMR (75 MHz, CDCl_3_): *δ* 21.6 (ArCH_3_), 23.0 (2 × CH_3_), 26.9 (CH), 68.9 (OCH_2_), 112.8 (Ar), 116.8 (=CH_2_), 121.6 (Ar), 126.8 (Ar), 134.0 (=CH), 134.5 (Ar), 136.4 (Ar), 156.0 (Ar). Anal. Calcd for C_13_H_18_O: C, 82.06; H, 9.54. Found: C, 82.03; H, 9.55.

*1-isopropyl-4-methyl-2-(prop-2-yn-1-yloxy)benzene* (**5**): colourless oil, 82% yield. ^1^H-NMR (300 MHz, CDCl_3_): *δ* 1.57–1.60 (m, 6H, 2 × CH_3_), 2.67 (s, 3H, ArCH_3_), 2.74 (t, *J* = 2.4 Hz, 1H, ≡CH), 3.67–3.70 (m, 1H, CH), 4.93–4.95 (m, 2H, OCH_2_), 7.09 (s, 1H, Ar), 7.13 (d, *J* = 8.4 Hz, 1H, Ar), 7.44–7.48 (m, 1H, Ar). ^13^C-NMR (75 MHz, CDCl_3_): *δ* 21.6 (ArCH_3_), 23.2 (2 × CH_3_), 26.8 (CH), 56.2 (OCH_2_), 75.5 (≡CH), 79.5 (C≡), 113.3 (Ar), 122.6 (Ar), 126.4 (Ar), 134.9 (Ar), 136.4 (Ar), 155.1 (Ar). Anal. Calcd for C_13_H_16_O: C, 82.94; H, 8.57. Found: C, 82.97; H, 8.60.

*2-(2-isopropyl-5-methylphenoxy)acetonitrile* (**6**): white sticky solid, 72% yield. ^1^H-NMR (300 MHz, CDCl_3_): *δ* 1.23–1.25 (m, 6H, 2 × CH_3_), 2.38 (s, 3H, ArCH_3_), 3.28–3.30 (m, 1H, CH), 4.77–4.78 (s, 2H, OCH_2_), 6.75 (s, 1H, Ar), 6.91 (d, *J* = 7.8 Hz, 1H, Ar), 7.19 (d, *J* = 7.5 Hz, 1H, Ar). ^13^C-NMR (75 MHz, CDCl_3_): *δ* 21.3 (ArCH_3_), 22.9 (2 × CH_3_), 26.5 (CH), 53.9 (OCH_2_), 112.9 (Ar), 115.5 (CN), 123.8 (Ar), 126.7 (Ar), 135.0 (Ar), 136.8 (Ar), 153.8 (Ar). Anal. Calcd for C_12_H_15_NO: C, 76.16; H, 7.99; N, 7.40. Found: C, 76.20; H, 8.03; N, 7.37.

*1-(2-isopropyl-5-methylphenoxy)propan-2-one* (**7**): amber-yellow oil, 90% yield. ^1^H-NMR (300 MHz, CDCl_3_): *δ* 1.30 (d, *J* = 7.2 Hz, 6H, 2 × CH_3_), 2.35–2.36 (m, 6H, CH_3_ + ArCH_3_), 3.29–3.43 (m, 1H, CH), 4.54 (s, 2H, OCH_2_), 6.55 (s, 1H, Ar), 6.84 (d, *J* = 7.8 Hz, 1H, Ar), 7.18 (d, *J* = 7.5, 1H, Ar). ^13^C-NMR (75 MHz, CDCl_3_): *δ* 21.3 (ArCH_3_), 22.8 (2 × CH_3_), 26.7 (CH_3_), 26.8 (CH), 73.2 (OCH_2_), 112.0 (Ar), 122.3 (Ar), 126.3 (Ar), 134.1 (Ar), 136.5 (Ar), 154.8(Ar), 206.6 (C=O). Anal. Calcd for C_13_H_18_O_2_: C, 75.69; H, 8.80. Found: C, 75.72; H, 8.77.

*2-(2-isopropyl-5-methylphenoxy)acetic acid***(8)**: white solid, 87% yield, mp = 143–145 °C. ^1^H-NMR (300 MHz, CDCl_3_): *δ* 1.23 (d, *J* = 6.9 Hz, 6H, 2 × CH_3_), 2.32 (s, 3H, ArCH_3_), 3.32–3.37 (m, 1H, CH), 4.69 (s, 2H, OCH_2_), 6.57 (s, 1H, Ar), 6.82 (d, *J* = 7.5 Hz, 1H, Ar), 7.14 (d, *J* = 7.5 Hz, 1H, Ar), 8.40 (bs, 1H, COOH). ^13^C-NMR (75 MHz, CDCl_3_): *δ* 21.3 (ArCH_3_), 22.8 (2 × CH_3_), 26.6 (CH), 65.2 (OCH_2_), 112.4 (Ar), 122.7 (Ar), 126.4 (Ar), 134.5 (Ar), 136.5 (Ar), 154.5 (Ar), 174.9 (CO). Anal. Calcd for C_12_H_16_O_3_: C, 69.21; H, 7.74. Found: C, 69.27; H, 7.77.

*2-butoxy-1-isopropyl-4-methylbenzene* (**9**): colourless oil, 75% yield. ^1^H-NMR (300 MHz, CDCl_3_) *δ* 1.27–1.32 (m, 3H, CH_3_), 1.53 (d, *J* = 6.3 Hz, 6H, 2 × CH_3_), 1.77–1.90 (m, 2H, CH_2_), 2.04–2.13 (m, 2H, CH_2_), 2.62 (s, 3H, ArCH_3_), 3.59–3.68 (m, 1H, CH), 4.21–4.25 (m, 2H, O CH_2_), 6.96 (s, 1H, Ar), 7.02 (d, *J* = 7.5 Hz, 1H, Ar), 7.39 (d, *J* = 7.5 Hz, 1H, Ar). ^13^C-NMR (75 MHz, CDCl_3_): *δ* 14.1 (CH_3_), 19.8 (CH_2_), 21.6 (ArCH_3_), 23.0 (2 × CH_3_), 26.9 (CH), 31.9 (CH_2_), 67.6 (OCH_2_), 112.3 (Ar), 121.1 (Ar), 126.0 (Ar), 134.2 (Ar), 136.4 (Ar), 156.5 (Ar). Anal. Calcd for C_14_H_22_O: C, 81.50; H, 10.75. Found: C, 81.55; H, 10.83.

*2-(but-2-en-1-yloxy)-1-isopropyl-4-methylbenzene* (**10**): pale yellow oil, 81% yield. ^1^H-NMR (300 MHz, CDCl_3_): *δ* 1.47 (d, *J* = 7.2 Hz, 6H, 2 × CH_3_), 1.98–2.00 (m, 3H, =CH_3_), 2.56 (s, 3H, ArCH_3_), 3.57–3.61 (m, 1H, CH), 4.66–4.69 (d, 2H, OCH_2_), 5.95–6.06 (m, 2H, 2 × =CH), 6.90 (s, 1H, Ar), 6.98 (d, *J* = 7.5 Hz, 1H, Ar), 7.34 (d, *J* = 7.8 Hz, 1H, Ar). ^13^C-NMR (75 MHz, CDCl_3_): *δ* 18.1 (CH_3_), 21.5 (ArCH_3_), 23.0 (2 × CH_3_), 26.8 (CH), 68.9 (OCH_2_), 112.9 (Ar), 121.4 (Ar), 126.1 (=CH), 127.0 (Ar), 129.1 (=CH), 134.5 (Ar), 136.3 (Ar), 156.1 (Ar). Mixture of *E*/*Z* isomers with ratio of 5.2/1. For sake of clarity, we have reported only the signals related to the major isomer. Anal. Calcd for C_14_H_20_O: C, 82.30; H, 9.87. Found: C, 82.33; H, 9.84.

*1-isopropyl-4-methyl-2-((3-methylbut-2-en-1-yl)oxy)benzene* (**11**): pale yellow oil, 78% yield. ^1^H-NMR (300 MHz, CDCl_3_): *δ* 1.38 (d, *J* = 7.2 Hz, 6H, 2 × CH_3_), 1.90–1.96 (m, 6H, 2 × CH_3_), 2.49 (s, 3H, ArCH_3_), 3.45–3.54 (m, 1H, CH), 4.68 (d, 2H, *J* = 6.6 Hz, OCH_2_), 5.66–5.71 (m, 1H, =CH), 6.85 (s, 1H, Ar), 6.91 (d, *J* = 8.1 Hz, 1H, Ar), 7.26 (d, *J* = 7.5 Hz, 1H, Ar). ^13^C-NMR (75 MHz, CDCl_3_): *δ* 18.3 (CH_3_), 21.5 (ArCH_3_), 23.0 (2 × CH_3_), 25.9 (CH_3_), 26.6 (CH), 65.2 (OCH_2_), 112.8 (Ar), 120.7 (-CH=), 121.2 (Ar), 126.0 (Ar), 134.5 (Ar), 136.2 (Ar), 137.0 (=C), 156.1 (Ar). Anal. Calcd for C_15_H_22_O: C, 82.52; H, 10.16. Found: C, 82.56; H, 10.12.

*1-isopropyl-4-methyl-2-(pentyloxy)benzene* (**12**): colourless oil, 88% yield. ^1^H-NMR (300 MHz, CDCl_3_): *δ* 1.30 (t, *J* = 6.9 Hz, 3H, CH_3_), 1.58 (d, *J* = 6.9 Hz, 6H, 2 × CH_3_), 1.71–1.85 (m, 4H, CH_2_), 2.09–2.19 (m, 2H, CH_2_), 2.66 (s, 3H, ArCH_3_), 3.63–3.72 (m, 1H, CH), 4.23–4.28 (m, 2H, OCH_2_), 6.99 (s, 1H, Ar), 7.06 (d, *J* = 7.5 Hz, 1H, Ar), 7.43 (d, *J* = 7.5 Hz, 1H, Ar). ^13^C-NMR (75 MHz, CDCl_3_): *δ* 14.4 (CH_3_), 21.6 (ArCH_3_), 22.8 (CH_2_), 23.1 (2 × CH_3_), 27.0 (CH), 28.8 (CH_2_), 29.5 (CH_2_), 68.0 (OCH_2_), 112.3 (Ar), 121.2 (Ar), 126.0 (Ar), 134.3 (Ar), 136.4 (Ar), 156.5 (Ar). Anal. Calcd for C_15_H_24_O: C, 81.76; H, 10.98. Found: C, 81.80; H, 11.02.

*ethyl 2-(2-isopropyl-5-methylphenoxy)acetate* (**13**): pale yellow oil, 69% yield. ^1^H-NMR (300 MHz, CDCl_3_): *δ* 1.36–1.40 (m, 9H, 3 × CH_3_), 2.41 (s, 3H, ArCH_3_), 3.52–3.55 (m, 1H, CH), 4.30–4.38 (m, 2H, OCH_2_CH_3_), 4.70 (s, 2H, OCH_2_), 6.66 (s, 1H, Ar), 6.89 (d, *J* = 7.5 Hz, 1H, Ar), 7.23 (d, *J* = 7.5 Hz, 1H, Ar). ^13^C-NMR (75 MHz, CDCl_3_) *δ* 14.2 (CH_3_), 21.3 (ArCH_3_), 22.8 (2 × CH_3_), 26.8 (CH), 61.1 (OCH_2_CH_3_), 65.6 (OCH_2_), 112.3 (Ar), 122.3 (Ar), 126.3 (Ar), 134.5 (Ar), 136.2 (Ar), 155.2 (Ar), 169.1 (C=O). Anal. Calcd for C_14_H_20_O_3_: C, 71.16; H, 8.53. Found: C, 71.20; H, 8.49.

*(E)-2-((3,7-dimethylocta-2,6-dien-1-yl)oxy)-1-isopropyl-4-methylbenzene* (**14**): colourless oil, 82% yield. ^1^H-NMR (300 MHz, CDCl_3_): *δ* 1.33 (d, *J* = 6.3 Hz, 6H, 2 × CH_3_), 1.74 (s, 3H, CH_3_), 1.81 (s, 3H, CH_3_), 1.85 (s, 3H, CH_3_), 2.19–2.27 (m, 4H, 2 × CH_2_), 2.44 (s, 3H, ArCH_3_), 3.42–3.44 (m, 1H, CH), 4.65 (d, *J* = 7.5 Hz, 2H, OCH_2_), 5.20–5.23 (m, 1H, =CH), 5.61–5.64 (m, 1H, =CH), 6.8 (s, 1H, Ar), 6.85 (d, *J* = 7.5 Hz, 1H, Ar), 7.21 (d, *J* = 7.8 Hz, 1H, Ar). ^13^C-NMR (75 MHz, CDCl_3_): *δ* 16.7 (CH_3_), 17.8 (CH_3_), 21.5 (ArCH_3_), 22.9 (2 × CH_3_), 25.8 (CH_3_), 26.5 (CH_2_), 26.6 (CH), 39.6 (CH_2_), 65.1 (OCH_2_), 112.8 (Ar), 120.5 (=CH), 121.1 (Ar), 124.0 (=CH), 125.9 (Ar), 131.7 (=C), 134.4 (Ar), 136.2 (Ar), 140.1 (=C), 156.1 (Ar). Mixture of *E*/*Z* isomers with ratio of 9.3/1. For sake of clarity, we have reported only the signals related to the major isomer. Calcd for C_20_H_30_O: C, 83.86; H, 10.56. Found: C, 83.80; H, 10.51.

*2-(benzyloxy)-1-isopropyl-4-methylbenzene* (**15**): pale yellow oil, 88% yield. ^1^H-NMR (300 MHz, CDCl_3_): *δ* 1.30 (d, *J* = 7.2 Hz, 6H, 2× CH_3_), 2.40 (s, 3H, ArCH_3_), 3.41–3.51 (m, 1H, CH), 5.13 (s, 2H, OCH_2_), 6.83 (s, 1H, Ar), 6.85 (d, *J* = 8.1 Hz, 1H, Ar), 7.21 (d, *J* = 7.8 Hz, 1H, Ar), 7.37–7.55 (m, 5H, Ar). ^13^C-NMR (75 MHz, CDCl_3_): *δ* 21.4 (ArCH_3_), 22.9 (2 × CH_3_), 26.6 (CH), 70.0 (OCH_2_), 112.7 (Ar), 121.5 (Ar), 126.0 (Ar), 127.2 (2 × Ar), 127.7 (Ar), 128.6 (2 × Ar), 134.4 (Ar), 136.4 (Ar), 137.7 (Ar), 155.9 (Ar). Anal. Calcd for C_17_H_20_O: C, 84.96; H, 8.39. Found: C, 84.90; H, 8.33.

*1-isopropyl-4-methyl-2-((2-methylbenzyl)oxy)benzene* (**16**): pale yellow oil, 88% yield. ^1^H-NMR (300 MHz, CDCl_3_): *δ* 1.58 (d, *J* = 7.2 Hz, 6H, 2 × CH_3_), 2.70 (s, 6H, 2 × ArCH_3_), 3.72-3.83 (m, 1H, CH), 5.32 (s, 2H, OCH_2_), 7.13–7.15 (m, 2H, Ar), 7.48–7.58 (m, 4H, Ar), 7.81–7.83 (m, 1H, Ar). ^13^C-NMR (75 MHz, CDCl_3_): *δ* 19.2 (ArCH_3_), 21.8 (ArCH_3_), 23.3 (2 × CH_3_), 26.8 (CH), 68.7 (OCH_2_), 112.7 (Ar), 121.8 (Ar), 126.3 (Ar), 126.4 (Ar), 128.3 (Ar), 128.5 (Ar), 135.0 (Ar), 134.6 (Ar), 135.8 (Ar), 136.6 (Ar), 136.6 (Ar), 156.2 (Ar). Anal. Calcd for C_18_H_22_O: C, 84.99; H, 8.72. Found: C, 84.95; H, 8.77.

*1-isopropyl-4-methyl-2-((4-methylbenzyl)oxy)benzene* (**17**): pale yellow oil, 77% yield. ^1^H-NMR (300 MHz, CDCl_3_): *δ* 1.68 (d, *J* = 6.9 Hz, 6H, 2 × CH_3_), 2.75 (s, 6H, 2 × ArCH_3_), 3.82–3.87 (m, 1H, CH), 5.38 (s, 2H, OCH_2_), 7.16 (s, 1H, Ar), 7.19 (d, *J* = 8.1 Hz, 1H, Ar), 7.54–7.59 (m, 3H, Ar), 7.75 (d, *J* = 8.4 Hz, 1H, Ar). ^13^C-NMR (75 MHz, CDCl_3_): *δ* 21.6 (ArCH_3_), 21.8 (ArCH_3_), 23.3 (2 × CH_3_), 27.1 (CH), 70.2 (OCH_2_), 113.0 (Ar), 121.8 (Ar), 126.3 (Ar), 127.7 (2 × Ar), 129.6 (2 × Ar), 128.6 (Ar), 135.0 (Ar), 136.6 (Ar), 137.7 (Ar), 156.3 (Ar). Anal. Calcd for C_18_H_22_O: C, 84.99; H, 8.72. Found: C, 84.93; H, 8.70.

*2-((3,5-dimethylbenzyl)oxy)-1-isopropyl-4-methylbenzene* (**18**): colourless oil, 67% yield. ^1^H-NMR (400 MHz, CDCl_3_): *δ* 1.28 (d, *J* = 6.9 Hz, 6H, 2 × CH_3_), 2.38 (s, 3H, ArCH_3_), 2.40 (bs, 6H, 2 × ArCH_3_), 3.40–3.47 (m, 1H, CH), 5.04 (s, 2H, OCH_2_), 6.80 (s, 1H, Ar), 6.82 (d, *J* = 7.9 Hz, 1H, Ar), 7.02 (s, 1H, Ar), 7.12 (s, 2H, Ar), 7.18 (d, *J* = 7.6 Hz, 1H, Ar). ^13^C-NMR (101 MHz, CDCl_3_): *δ* 21.3 (2 × ArCH_3_), 21.4 (ArCH_3_), 22.9 (2 × CH_3_), 26.6 (CH), 70.2 (OCH_2_), 112.8 (Ar), 121.4 (Ar), 125.0 (2 × Ar), 125.9 (Ar), 129.4 (Ar), 134.4 (Ar), 136.4 (Ar), 137.6 (Ar), 138.1 (2 × Ar), 156.0 (Ar). Anal. Calcd for C_19_H_24_O_3_: C, 85.03; H, 9.01. Found: C, 85.08; H, 9.00.

*1-isopropyl-2-((3-methoxybenzyl)oxy)-4-methylbenzene* (**19**): colourless oil, 82% yield. ^1^H-NMR (400 MHz, CDCl_3_): *δ* 1.28 (d, *J* = 6.9 Hz, 6H, 2 × CH_3_), 2.37 (s, 3H, ArCH_3_), 3.38–3.48 (m, 1H, CH), 3.87 (s, 3H, OCH_3_), 5.09 (s, 2H, OCH_2_), 6.79 (s, 1H, Ar), 6.82 (d, *J* = 7.8 Hz, 1H, Ar), 6.90–6.92 (m, 1H, Ar), 7.07–7.08 (m, 2H, Ar), 7.17 (d, *J* = 7.7 Hz, 1H, Ar), 7.35 (t, *J* = 8.1 Hz, 1H, Ar). ^13^C-NMR (101 MHz, CDCl_3_): *δ* 21.4 (ArCH_3_), 22.8 (2 × CH_3_), 26.7 (CH), 55.5 (OCH_3_), 69.8 (OCH_2_), 112.5 (Ar), 112.7 (Ar), 113.2 (Ar), 119.3 (Ar), 121.5 (Ar), 126.0 (Ar), 129.6 (Ar), 134.4 (Ar), 136.4 (Ar), 139.3 (Ar), 155.8 (Ar). Anal. Calcd for C_18_H_22_O_2_: C, 79.96; H, 8.20. Found: C, 79.90; H, 8.25.

*1-isopropyl-4-methyl-2-((2-(trifluoromethyl)benzyl)oxy)benzene* (**20**): colourless oil, 77% yield. ^1^H-NMR (300 MHz, CDCl_3_): *δ* 1.39–1.42 (m, 6H, 2 × CH_3_), 2.46 (s, 3H, ArCH_3_), 3.55–3.57 (m, 1H, CH), 5.42 (s, 2H, OCH_2_), 6.85 (s, 1H, Ar), 6.94 (d, *J* = 7.5 Hz, 1H, Ar), 7.30 (d, *J* = 7.5 Hz, 1H, Ar), 7.51–7.54 (m, 1H, Ar), 7.68–7.71 (m, 1H, Ar), 7.82 (d, *J* = 7.5 Hz, 1H, Ar), 7.96 (d, *J* = 7.8 Hz, 1H, Ar). ^13^C-NMR (75 MHz, CDCl_3_): *δ* 21.4 (ArCH_3_), 22.9 (2 × CH_3_), 26.7 (CH), 66.3 (t, ^4^*J_C-F_* = 3.4 Hz, OCH_2_), 112.7 (Ar), 121.9 (Ar), 124.6 (d, ^1^*J_C-F_* = 272.3 Hz, CF_3_), 125.9 (q, ^2^*J_C-F_* = 57 Hz, Ar), 126.2 (Ar) 127.2 (d, ^3^*J_C-F_* = 30.9 Hz, Ar), 127.6 (Ar), 128.4 (Ar), 132.3 (Ar), 134.4 (Ar), 136.5 (Ar), 136.6 (Ar), 155.5 (Ar). ^19^F NMR (564.7 MHz, CDCl_3_) *δ−*58.69 (s, 3F, CF_3_). Anal. Calcd for C_18_H_19_F_3_O: C, 70.12; H, 6.21. Found: C, 70.08; H, 6.23. 

*1-isopropyl-4-methyl-2-((3-(trifluoromethyl)benzyl)oxy)benzene* (**21**): colourless oil, 69% yield. ^1^H-NMR (300 MHz, CDCl_3_): *δ* 1.59–1.63 (m, 6H, 2 × CH_3_), 2.65–2.66 (m, 3H, ArCH_3_), 3.74–3.77 (m, 1H, CH), 5.35 (s, 2H, OCH_2_), 7.06 (s, 1H, Ar), 7.13 (d, *J* = 7.5 Hz, 1H, Ar), 7.48–7.51 (m, 1H, Ar), 7.75–7.78 (m, 1H, Ar), 7.87 (d, *J* = 8.4 Hz, 1H, Ar), 7.93 (d, *J* = 7.5 Hz, 1H, Ar), 8.08 (s, 1H, Ar). ^13^C-NMR (75 MHz, CDCl_3_): *δ* 21.5 (ArCH_3_), 23.0 (2 × CH_3_), 27.0 (CH), 69.3 (OCH_2_), 112.9 (Ar), 121.0 (d, ^1^*J_C-F_* = 274.6 Hz, CF_3_), 122.2 (Ar), 124.0 (m, Ar), 124.7 (q, ^4^*J_C-F_* = 3.4 Hz, Ar), 126.4 (Ar), 129.3 (Ar), 130.5 (Ar), 131.2 (d, ^3^*J_C-F_* = 32.0 Hz, Ar), 134.6 (Ar), 136.7 (Ar), 139.1 (Ar), 155.8 (Ar). ^19^F NMR (564.7 MHz, CDCl_3_) *δ−*60.98 (s, 3F, CF_3_). Anal. Calcd for C_18_H_19_F_3_O: C, 70.12; H, 6.21. Found: C, 70.10; H, 6.20. 

*1-isopropyl-4-methyl-2-((4-(trifluoromethyl)benzyl)oxy)benzene* (**22**): colourless oil, 87% yield. ^1^H-NMR (300 MHz, CDCl_3_): *δ* 1.46 (d, *J* = 7.2 Hz, 6H, 2 × CH_3_), 2.51 (s, 3H, ArCH_3_), 3.52–3.61 (m, 1H, CH), 5.27 (s, 2H, OCH_2_), 6.900 (s, 1H, Ar), 6.99 (d, *J* = 8.4 Hz, 1H, Ar), 7.35 (d, *J* = 7.5 Hz, 1H, Ar), 7.73 (d, *J* = 7.5 Hz, 2H, Ar), 7.83 (d, *J* = 8.7 Hz, 1H, Ar). ^13^C-NMR (75 MHz, CDCl_3_): *δ* 21.4 (ArCH_3_), 22.9 (2 × CH_3_), 26.8 (CH), 69.1 (OCH_2_), 112.7 (Ar), 120.8 (d, ^1^*J_C-F_* = 273.4 Hz, CF_3_), 122.0 (Ar), 125.6 (q, ^4^*J_C-F_* = 3.4 Hz, Ar), 126.3 (Ar) 127.2 (2 × Ar), 129.8 (m, 2 × Ar), 134.4 (Ar), 136.6 (Ar), 141.9 (Ar), 155.6 (Ar). ^19^F NMR (564.7 MHz, CDCl_3_) *δ−*60.80 (s, 3F, CF_3_). Anal. Calcd for C_18_H_19_F_3_O: C, 70.12; H, 6.21. Found: C, 70.16; H, 6.22. 

*2-((3,5-bis(trifluoromethyl)benzyl)oxy)-1-isopropyl-4-methylbenzene* (**23**): colourless oil, 80% yield. ^1^H-NMR (400 MHz, CDCl_3_): *δ* 1.27 (d, *J* = 6.9 Hz, 6H, 2 × CH_3_), 2.37 (s, 3H, ArCH_3_), 3.33–3.43 (m, 1H, CH), 5.20 (s, 2H, OCH_2_), 6.75 (s, 1H, Ar), 6.86 (d, *J* = 7.7 Hz, 1H, Ar), 7.19 (d, *J* = 7.7 Hz, 1H, Ar), 7.89 (s, 1H, Ar), 7.96 (s, 2H, Ar). ^13^C-NMR (101 MHz, CDCl_3_): *δ* 21.3 (ArCH_3_), 22.8 (2 × CH_3_), 26.7 (CH), 68.6 (OCH_2_), 112.7 (Ar), 119.2 (Ar), 121.6–121.8 (m, Ar), 122.3 (Ar), 123.3 (d, ^1^*J_C-F_* = 272.5 Hz, 2 × CF_3_), 126.3 (Ar), 126.9 (Ar), 127.0 (Ar), 127.4 (Ar), 131.9 (q, ^2^*J_C-F_* = 33.4 Hz, Ar), 134.4 (Ar), 136.6 (Ar), 140.3 (Ar), 155.1 (Ar). ^19^F NMR (564.7 MHz, CDCl_3_) *δ−*66.85 (s, 3F, CF_3_). Anal. Calcd for C_19_H_18_F_6_O: C, 60.64; H, 4.82. Found: C, 60.60; H, 4.77.

*4-((2-isopropyl-5-methylphenoxy)methyl)benzonitrile* (**24**): pale yellow solid, 71% yield, mp = 76–78 °C. ^1^H-NMR (300 MHz, CDCl_3_): *δ* 1.34–1.36 (m, 6H, 2 × CH_3_), 2.41 (s, 3H, ArCH_3_), 3.46–3.50 (m, 1H, CH), 5.19 (s, 2H, OCH_2_), 6.79 (s, 1H, Ar), 6.89 (d, *J* = 7.5 Hz, 1H, Ar), 7.24 (d, *J* = 7.5 Hz, 1H, Ar), 7.63 (d, *J* = 7.8 Hz, 2H, Ar), 7.72 (d, *J* = 8.1 Hz, 2H, Ar). ^13^C-NMR (75 MHz, CDCl_3_): *δ* 21.4 (ArCH_3_), 22.9 (2 × CH_3_), 26.7 (CH), 68.9 (OCH_2_), 111.5 (Ar), 112.6 (Ar), 118.9 (CN), 122.1 (Ar), 126.2 (Ar), 127.4 (2 × Ar), 132.4 (2 × Ar), 134.3 (Ar), 136.5 (Ar), 143.2 (Ar), 155.3 (Ar). Anal. Calcd for C_18_H_19_NO: C, 81.47; H, 7.22; N, 5.28. Found: C, 81.50; H, 7.20; N, 5.31.

*2-((3-fluorobenzyl)oxy)-1-isopropyl-4-methylbenzene* (**25**): colourless oil, 82% yield. ^1^H-NMR (300 MHz, CDCl_3_): *δ* 1.59–1.61 (d, *J* = 6.3 Hz, 6H, 2 × CH_3_), 2.65 (s, 3H, ArCH_3_), 3.71–3.81 (m, 1H, CH), 5.30 (s, 2H, OCH_2_), 7.03 (s, 1H, Ar), 7.12 (d, *J* = 8.4 Hz, 1H, Ar), 7.25–7.31 (m, 1H, Ar), 7.47–7.52 (m, 3H, Ar), 7.57–7.64 (m, 1H, Ar). ^13^C-NMR (75 MHz, CDCl_3_): *δ* 21.6 (ArCH_3_), 23.1 (2 × CH_3_), 27.0 (CH), 69.3 (OCH_2_), 112.8 (Ar), 114.2 (d, ^2^*J*_C-F_ = 21.7 Hz, Ar), 114.8 (d, ^2^*J*_C-F_ = 21.7 Hz, Ar), 122.1 (Ar), 122.7 (d, ^4^*J*_C-F_ = 2.3 Hz, Ar), 126.4 (Ar), 130.3 (d, ^3^*J*_C-F_ = 8.0 Hz, Ar), 134.6 (Ar), 136.6 (Ar), 140.6 (d, ^3^*J*_C-F_ = 6.8 Hz, Ar), 155.9 (Ar), 163.3 (d, ^1^*J*_C-F_ = 244.9 Hz, Ar-F). ^19^F NMR (564.7 MHz, CDCl_3_) *δ−*111.18 (ddd, 1F, *J*_F–H_ = 7.91 Hz (ortho), 6.2 Hz (meta), CF). Anal. Calcd for C_17_H_19_FO: C, 79.04; H, 7.41. Found: C, 79.10; H, 7.50.

*1,3-difluoro-2-((2-isopropyl-5-methylphenoxy)methyl)benzene* (**26**): white solid, 83% yield, mp = 91–92 °C. ^1^H-NMR (300 MHz, CDCl_3_): *δ* 1.22 (d, *J* = 7.2 Hz, 6H, 2 × CH_3_), 2.42 (s, 3H, ArCH_3_), 3.26–3.35 (m, 1H, CH), 5.17 (s, 2H, OCH_2_), 6.86 (d, *J* = 7.5 Hz, 1H, Ar), 6.93–7.03 (m, 3H, Ar), 7.18 (d, *J* = 8.1 Hz, 1H, Ar), 7.31–7.41 (m, 2H, Ar). ^13^C-NMR (75 MHz, CDCl_3_): *δ* 21.4 (ArCH_3_), 22.8 (2 × CH_3_), 26.5 (CH), 58.2 (OCH_2_), 111.3 (2 × Ar), 111.6 (Ar), 113.0 (Ar), 121.9 (Ar), 126.1 (Ar), 130.5 (t, ^3^*J*_C-F_ = 10.3 Hz, Ar), 134.7 (Ar), 136.4 (Ar), 155.7 (Ar), 162.0 (d, ^1^*J*_C-F_ = 248.3 Hz, Ar-F), 162.1 (d, ^1^*J*_C-F_ = 249.5 Hz, Ar-F). ^19^F NMR (564.7 MHz, CDCl_3_) *δ−*112.97 (t, 2F, *J*_F–H_ = 7.5 Hz, CF). Anal. Calcd for C_17_H_18_F_2_O: C, 73.89; H, 6.57. Found: C, 73.93; H, 6.52.

*2-((3,5-difluorobenzyl)oxy)-1-isopropyl-4-methylbenzene* (**27**): white solid, 90% yield, mp = 35–36 °C. ^1^H-NMR (400 MHz, CDCl_3_): *δ* 1.27 (d, *J* = 6.9 Hz, 6H, 2 × CH_3_), 2.35 (s, 3H, ArCH_3_), 3.35–3.45 (m, 1H, CH), 5.08 (s, 2H, OCH_2_), 6.70 (s, 1H, Ar), 6.76–6.84 (m, 2H, Ar), 6.99–7.03 (m, 2H, Ar), 7.18 (d, *J* = 7.7 Hz, 1H, Ar). ^13^C-NMR (101 MHz, CDCl_3_): *δ* 21.3 (ArCH_3_), 22.8 (2 × CH3), 26.6 (CH), 68.7 (OCH_2_), 103.0 (t, ^2^*J_C-F_* = 25.3 Hz, Ar), 109.5 (d, ^2^*J_C-F_* = 25.7 Hz, 2 × Ar), 112.6 (Ar), 121.9 (Ar), 126.2 (Ar), 134.4 (Ar), 136.5 (Ar), 141.8 (t, ^3^*J_C-F_* = 9.1 Hz, Ar), 155.2 (Ar), 163.1 (d, ^1^*J_C-F_* = 248.5 Hz, C-F), 163.2 (d, ^1^*J_C-F_* = 248.6 Hz, C-F). ^19^F NMR (564.7 MHz, CDCl_3_) *δ−*113.39 (t, 2F, *J*_F–H_ = 7.5 Hz, CF). Anal. Calcd for C_17_H_18_F_2_O: C, 73.89; H, 6.57. Found: C, 73.93; H, 6.61.

*2-((3-chlorobenzyl)oxy)-1-isopropyl-4-methylbenzene* (**28**): yellow sticky oil, 91% yield. ^1^H-NMR (400 MHz, CDCl_3_): *δ* 1.28 (d, *J* = 6.8 Hz, 6H, 2 × CH_3_), 2.37 (s, 3H, ArCH_3_), 3.40–3.44 (m, 1H, CH), 5.08 (s, 2H, OCH_2_), 6.76 (s, 1H, Ar), 6.84 (d, *J* = 7.6 Hz, 1H, Ar), 7.19 (d, *J* = 7.6 Hz, 1H, Ar), 7.35–7.38 (m, 3H, Ar), 7.49 (s, 1H, Ar). ^13^C-NMR (101 MHz, CDCl_3_): *δ* 21.4 (ArCH_3_), 22.9 (2 × CH_3_), 26.6 (CH), 69.2 (OCH_2_), 112.7 (Ar), 121.8 (Ar), 125.1 (Ar), 126.1 (Ar), 127.1 (Ar), 127.9 (Ar), 129.8 (Ar), 134.4 (Ar), 136.4 (Ar), 139.8 (Ar), 155.6 (Ar). Anal. Calcd for C_17_H_19_ClO: C, 74.31; H, 6.97. Found: C, 74.27; H, 6.90.

*2-((4-chlorobenzyl)oxy)-1-isopropyl-4-methylbenzene* (**29**): colourless oil, 74% yield. ^1^H-NMR (300 MHz, CDCl_3_): *δ* 1.43–1.46 (m, 6H, 2 × CH3), 2.52 (s, 3H, ArCH_3_), 3.55–3.58 (m, 1H, CH), 5.17 (s, 2H, OCH_2_), 6.90 (s, 1H, Ar), 6.98 (d, *J* = 7.5 Hz, 1H, Ar), 7.33 (d, *J* = 7.8 Hz, 1H, Ar), 7.50–7.53 (m, 4H, Ar). ^13^C-NMR (75 MHz, CDCl_3_) *δ* 21.6 (ArCH_3_), 23.0 (2 × CH_3_), 26.9 (CH), 69.3 (OCH_2_), 112.8 (Ar), 121.9 (Ar), 126.2 (Ar), 128.6 (2 × Ar), 128.9 (2 × Ar), 133.6 (Ar), 134.4 (Ar), 136.3 (Ar), 136.5 (Ar), 155.8 (Ar). Anal. Calcd for C_17_H_19_ClO: C, 74.31; H, 6.97. Found: C, 74.37; H, 7.01.

*2-chloro-1-((2-isopropyl-5-methylphenoxy)methyl)-4-methoxybenzene* (**30**): white solid, 71% yield, mp = 84–86 °C. ^1^H-NMR (400 MHz, CDCl_3_): *δ* 1.25 (d, *J* = 6.9 Hz, 6H, 2 × CH3), 2.36 (s, 3H, ArCH_3_), 3.34–3.44 (m, 1H, CH), 3.85 (s, 3H, OCH_3_), 5.11 (s, 2H, OCH_2_), 6.79 (s, 1H, Ar), 6.81 (d, *J* = 7.9 Hz, 1H, Ar), 6.89 (dd, *J* = 8.6 Hz, 2.5 Hz, 1H, Ar), 7.00 (d, *J* = 2.5 Hz, 1H, Ar), 7.16 (d, *J* = 7.6 Hz, 1H, Ar), 7.51 (d, *J* = 8.6 Hz, 1H, Ar). ^13^C-NMR (101 MHz, CDCl_3_): *δ* 21.4 (ArCH_3_), 22.8 (2 × CH3), 26.6 (CH), 55.6 (OCH_3_), 67.1 (OCH_2_), 112.7 (Ar), 112.9 (Ar), 114.9 (Ar), 121.6 (Ar), 126.0 (Ar), 127.3 (Ar), 129.8 (Ar), 133.5 (Ar), 134.4 (Ar), 136.4 (Ar), 155.5 (Ar), 159.7 (Ar). Anal. Calcd for C_18_H_21_ClO_2_: C, 70.93; H, 6.94. Found: C, 70.88; H, 6.96.

*2-((2-bromobenzyl)oxy)-1-isopropyl-4-methylbenzene* (**31**): white sticky solid, 71% yield. ^1^H-NMR (300 MHz, CDCl_3_): *δ* 1.47 (d, *J* = 7.2 Hz, 6H, 2 × CH3), 2.52 (s, 3H, ArCH_3_), 3.59–3.66 (m, 1H, CH), 5.29 (s, 2H, OCH_2_), 6.94 (s, 1H, Ar), 6.98 (d, *J* = 7.5 Hz, 1H, Ar), 7.30–7.35 (m, 2H, Ar), 7.49–7.54 (m, 1H, Ar), 7.73–7.76 (m, 1H, Ar), 7.80 (d, *J* = 6.9 Hz, 1H, Ar). ^13^C-NMR (75 MHz, CDCl_3_): *δ* 21.4 (ArCH_3_), 22.9 (2 × CH3), 26.6 (CH), 70.0 (OCH_2_), 112.7 (Ar), 121.5 (Ar), 126.0 (Ar), 127.2 (Ar), 127.7 (Ar), 128.6 (Ar), 134.4 (Ar), 136.4 (Ar), 137.7 (Ar), 155.9 (Ar). Anal. Calcd for C_17_H_19_BrO: C, 63.69; H, 6.00. Found: C, 63.71; H, 5.57.

*2-((4-bromobenzyl)oxy)-1-isopropyl-4-methylbenzene* (**32**): colourless oil, 71% yield. ^1^H-NMR (300 MHz, CDCl_3_): *δ* 1.52 (d, *J* = 6.9 Hz, 6H, 2 × CH3), 2.58 (s, 3H, ArCH_3_), 3.61–3.70 (m, 1H, CH), 5.20 (s, 2H, OCH_2_), 6.96 (s, 1H, Ar), 7.05 (d, *J* = 7.5 Hz, 1H, Ar), 7.40 (d, *J* = 7.5 Hz, 1H, Ar), 7.52–7.54 (d, 2H, Ar), 7.70–7.74 (m, 2H, Ar). ^13^C-NMR (75 MHz, CDCl_3_): *δ* 21.7 (ArCH_3_), 23.1 (2 × CH3), 27.0 (CH), 69.4 (OCH_2_), 112.8 (Ar), 121.8 (Ar), 122.0 (Ar), 126.3 (Ar), 129.0 (2 × Ar), 131.9 (2 × Ar), 134.5 (Ar), 136.5 (Ar), 136.9 (Ar), 155.8 (Ar). Anal. Calcd for C_17_H_19_BrO: C, 63.69; H, 6.00. Found: C, 63.65; H, 6.07.

*2-((4-iodobenzyl)oxy)-1-isopropyl-4-methylbenzene* (**33**): yellowish sticky oil, 83% yield. ^1^H-NMR (400 MHz, CDCl_3_): *δ* 1.26 (d, *J* = 6.9 Hz, 6H, 2 × CH3), 2.36 (s, 3H, ArCH_3_), 3.33–3.43 (m, 1H, CH), 5.04 (s, 2H, OCH_2_), 6.74 (s, 1H, Ar), 6.82 (d, *J* = 7.7 Hz, 1H, Ar), 7.17 (d, *J* = 7.7 Hz, 1H, Ar), 7.23 (d, *J* = 8.2 Hz, 2H, Ar), 7.76 (d, *J* = 8.3 Hz, 1H, Ar). ^13^C-NMR (101 MHz, CDCl_3_): *δ* 21.4 (ArCH_3_), 22.8 (2 × CH3), 26.6 (CH), 69.3 (OCH_2_), 93.1 (Ar), 112.6 (Ar), 121.7 (Ar), 126.1 (Ar), 129.0 (2 × Ar), 134.3 (Ar), 136.4 (Ar), 137.4 (Ar), 137.6 (2 × Ar), 155.6 (Ar). Anal. Calcd for C_17_H_19_IO: C, 55.75; H, 5.23. Found: C, 55.80; H, 5.30.

*1-isopropyl-4-methyl-2-((2-nitrobenzyl)oxy)benzene* (**34**): brown solid, 77% yield, mp = 77–79 °C. ^1^H-NMR (300 MHz, CDCl_3_): *δ* 1.26 (d, *J* = 7.2 Hz, 6H, 2× CH_3_), 2.32 (s, 3H, ArCH_3_), 3.38–3.43 (m, 1H, CH), 5.48 (s, 2H, OCH_2_), 6.73 (s, 1H, Ar), 6.81 (d, *J* =7.5 Hz, 1H, Ar), 7.16 (d, *J* =7.5, 1H, Ar), 7.50–7.53 (m, 1H, Ar), 7.69–7.75 (m, 1H, Ar), 7.96 (d, *J* = 7.8 Hz, 1H, Ar), 8.20 (dd, *J* = 8.1 Hz, *J* = 1.2 Hz, 1H, Ar). Anal. Calcd for C_17_H_19_NO_3_: C, 71.56; H, 6.71; N, 4.91. Found: C, 71.60; H, 6.75; N, 4.88.

*1-isopropyl-4-methyl-2-((3-nitrobenzyl)oxy)benzene* (**35**): amber-yellow oil, 81% yield. ^1^H-NMR (300 MHz, CDCl_3_): *δ* 1.48–1.52 (m, 6H, 2 × CH3), 2.53 (s, 3H, ArCH_3_), 3.62–3.67 (m, 1H, CH), 5.27 (s, 2H, OCH_2_), 6.95 (s, 1H, Ar), 7.00 (d, *J* = 7.8 Hz, 1H, Ar), 7.33–7.37 (m, 1H, Ar), 7.65–7.71 (m, 1H, Ar), 7.97 (d, *J* = 6.9 Hz, 1H, Ar), 8.26–8.29 (m, 1H, Ar), 8.51 (s, 1H, Ar). ^13^C-NMR (75 MHz, CDCl_3_): *δ* 21.5 (ArCH_3_), 23.0 (2 × CH3), 27.0 (CH), 68.6 (OCH_2_), 112.7 (Ar), 121.8 (Ar), 122.3 (Ar), 122.7 (Ar), 126.3 (Ar), 129.7 (Ar), 133.0 (Ar), 134.3 (Ar), 136.6 (Ar), 140.1 (Ar), 148.5 (Ar), 155.5 (Ar). Anal. Calcd for C_17_H_19_NO_3_: C, 71.56; H, 6.71; N, 4.91. Found: C, 71.55; H, 6.73; N, 4.91.

*1-isopropyl-4-methyl-2-((4-nitrobenzyl)oxy)benzene* (**36**): white solid, 81% yield, mp = 81–83 °C. ^1^H-NMR (300 MHz, CDCl_3_): *δ* 1.24–1.26 (m, 6H, 2 × CH3), 2.33 (s, 3H, ArCH_3_), 3.36–3.40 (m, 1H, CH), 5.18 (s, 2H, OCH_2_), 6.70 (s, 1H, Ar), 6.82 (d, *J* = 7.5 Hz, 1H, Ar), 7.16 (d, *J* = 7.8 Hz, 1H, Ar), 7.63 (d, *J* = 8.4 Hz, 2H, Ar), 8.26–8.28 (m, 2H, Ar). ^13^C-NMR (75 MHz, CDCl_3_): *δ* 21.4 (ArCH_3_), 22.8 (2 × CH3), 26.7 (CH), 68.7 (OCH_2_), 112.5 (Ar), 122.0 (Ar), 123.9 (2 × Ar), 126.2 (Ar), 127.3 (2 × Ar), 134.3 (Ar), 136.5 (Ar), 145.1 (Ar), 147.4 (Ar), 155.1 (Ar). Anal. Calcd for C_17_H_19_NO_3_: C, 71.56; H, 6.71; N, 4.91. Found: C, 71.51; H, 6.67; N, 4.96.

*1-((2-isopropyl-5-methylphenoxy)methyl)naphthalene* (**37**): pale yellow oil, 76% yield. ^1^H-NMR (400 MHz, CDCl_3_): *δ* 1.21 (d, *J* = 6.9 Hz, 6H, 2 × CH3), 2.41 (s, 3H, ArCH_3_), 3.31–3.42 (m, 1H, CH), 5.53 (m, 2H, CH_2_O), 6.86 (d, *J* = 7.7 Hz, 1H, Ar), 6.95 (s, 1H, Ar), 7.20 (d, *J* = 7.7 Hz, 1H, Ar), 7.51–7.58 (m, 3H, Ar), 7.68 (d, *J* = 6.9 Hz, 1H, Ar), 7.90 (d, *J* = 8.2 Hz, 1H, Ar), 7.93–7.96 (m, 1H, Ar), 8.09–8.11 (m, 1H, Ar). ^13^C-NMR (101 MHz, CDCl_3_): *δ* 21.5 (ArCH_3_), 23.0 (2 × CH3), 26.4 (CH), 68.6 (OCH_2_), 112.6 (Ar), 121.6 (Ar), 123.8 (Ar), 125.4 (Ar), 125.9 (Ar), 126.0 (Ar), 126.1 (Ar), 126.3 (Ar), 128.7 (Ar), 128.8 (Ar), 131.5 (Ar), 133.0 (Ar), 133.7 (Ar), 134.5 (Ar), 136.4 (Ar), 155.9 (Ar). Anal. Calcd for C_21_H_22_O: C, 86.85; H, 7.64. Found: C, 86.84; H, 7.70.

*4-((2-isopropyl-5-methylphenoxy)methyl)-1,1′-biphenyl* (**38**): white solid, 78% yield, mp = 91–92 °C. ^1^H-NMR (400 MHz, CDCl_3_): *δ* 1.29 (d, *J* = 6.9 Hz, 6H, 2 × CH3), 2.38 (s, 3H, ArCH_3_), 3.40–3.50 (m, 1H, CH), 5.16 (s, 2H, OCH_2_), 6.83–6.84 (m, 2H, Ar), 7.19 (d, *J* = 7.8 Hz, 1H, Ar), 7.38–7.42 (m, 1H, Ar), 7.47–7.52 (m, 2H, Ar), 7.57 (d, *J* = 8.3 Hz, 1H, Ar), 7.64–7.68 (m, 4H, Ar). ^13^C-NMR (101 MHz, CDCl_3_): *δ* 21.4 (ArCH_3_), 22.9 (2 × CH3), 26.6 (CH), 69.7 (OCH_2_), 112.7 (Ar), 121.5 (Ar), 127.1 (2 × Ar), 127.3 (2 × Ar), 127.4 (Ar), 127.6 (2 × Ar), 128.8 (2 × Ar), 134.4 (Ar), 136.4 (Ar), 137.4 (Ar), 136.7 (Ar), 140.7 (Ar), 140.9 (Ar), 155.8 (Ar). Anal. Calcd for C_23_H_24_O: C, 87.30; H, 7.64. Found: C, 87.35; H, 7.67.

*2-((2-isopropyl-5-methylphenoxy)methyl)isoindoline-1,3-dione* (**39**): white solid, 79% yield, mp = 121–122 °C. ^1^H-NMR (300 MHz, CDCl_3_): *δ* 1.18 (d, *J* = 7.2 Hz, 6H, 2 × CH3), 2.36 (s, 3H, ArCH_3_); 3.24–3.33 (m, 1H, CH_3_), 5.67 (s, 2H, OCH_2_), 6.84 (d, *J* = 7.8 Hz, 1H, Ar), 7.02 (s, 1H, Ar), 7.12 (d, *J* = 7.8 Hz, 1H, Ar), 7.73–7.78 (m, 2H, Ar), 7.88–7.92 (m, 2H, Ar). ^13^C-NMR (75 MHz, CDCl_3_): *δ* 21.3 (ArCH_3_), 23.0 (2 × CH3), 26.5 (CH), 65.7 (OCH_2_), 115.0 (Ar), 123.2 (2 × Ar), 123.8 (2 × Ar), 126.3 (Ar), 131.8 (Ar), 134.5 (2 × Ar), 135.4 (Ar), 136.5 (Ar), 153.4 (Ar), 167.2 (2 × C=O). Anal. Calcd for C_19_H_19_NO_3_: C, 73.77; H, 6.19; N, 4.53. Found: C, 73.71; H, 6.24; N, 4.50.

### 3.4. Anti-Helicobacter pylori Activity

The MIC determination was performed by modified broth microdilution assay as previously described [24]. For MBC evaluation, 10 μL of suspensions without visible growth were spotted on Skirrow agar plates surface and incubated for 72 h at 37 °C in microaerophilic conditions and 100% humidity conditions. The MBC was defined as the concentration that killed 99.9% of the initial inoculum.

### 3.5. Cell Lines and Treatments

The human adenocarcinoma gastric cell line (AGS) was derived from an untreated human adenocarcinoma of the stomach and retained the same cytological characteristics of the malignant cells obtained from Caucasian patients [25]. The AGS cells (ECACC 89090402) were purchased from CLS Cell Lines Services GmbH (Epplheim, Germany) and cultured in Dulbecco’s Modified Eagle’s Medium (DMEM) supplemented with 4.5 g/L glucose, 2 mM L-glutamine, and 10% Foetal Bovine Serum (FBS) (EuroClone S.p.A., Pero, Italy). Working solutions of thymol (**1**) and its derivatives (**9**, **15**, **24**–**26**, **30**, **34**–**36**, **38**, **39**) (600 mM) were freshly prepared in dimethyl sulfoxide (DMSO) and DMEM according to the experimental design by serial dilutions in complete culture medium. The final concentration of DMSO in experiments was 0.14%. No toxicity on AGS cells was observed (data not shown). 5-Fluorouracil and carvacrol (Sigma-Aldrich, Milan, Italy) were used as positive controls.

### 3.6. Cell Viability

Cell viability was evaluated by MTS (3-(4,5-dimethylthiazol-2-yl)-5-(3-carboxymethoxyphenyl)-2-(4-sulfophenyl)-2*H*-tetrazolium)) assay (Promega, Madison, WI, USA). The concentration ranges of thymol and its derivatives for the treatment was extrapolated from concentration-response curves built in preliminary experiments. Briefly, AGS cells were seeded in 96-well plates (6 × 10^3^ cells/well) and treated for 24 h at 37 °C in a humidified atmosphere with 5% CO_2_ with several concentrations (50–800 μM) of each compound (5 replica wells for each treatment condition). Cells were incubated with the MTS solution for at least 1 h and cell viability was tested colorimetrically by measuring the absorbance at 490 nm using GloMax-Multi Detection System (Promega, Madison, WI, USA). Cell viability was reported as the percentage as compared with the untreated cells recognized as 100%. The IC_50_ value was determined from the concentration-response curves by nonlinear regression analysis [15].

### 3.7. Statistical Analysis

A *p* value of 0.05 was assessed as statistically significant. IC_50_ values were obtained using the GraphPad Prism 7 software (GraphPad Software, San Diego, CA, USA).

## 4. Conclusions

We have explored a thymol-based molecular library with large structural diversity to discover new dual-action agents characterized by the ability to reduce the growth of several strains of *H. pylori* and to show toxicity for AGS cells. This approach allowed us to demonstrate, as also reported for carvacrol analogues, that the antibacterial activity of these phenolic terpenes is not limited to the presence of the free OH group in the natural parent compounds and that their antimicrobial potential could be enlarged by chemical modifications. Indeed, the proper functionalization could improve the anti-*Helicobacter pylori* activity especially against strains endowed with a different susceptibility pattern to antibiotics currently used in therapy. Moreover, some derivatives could display an antiproliferative effect useful to assess them as dual-acting agents for contrasting the development of gastric cancer. Further studies will address the potential of these compounds to treat biofilm-associated *H. pylori* infections in vitro and in vivo due to the disaggregating ability of their parent compound [22] and the putative mechanism of action, which could be alternative to those reported in literature for the parent compound [26]. Future studies will be devoted to the role of these compounds on the activity and role of carbonic anhydrase in *H. pylori* [27].

## Figures and Tables

**Table 1:** MIC and MBC values for thymol (**1**, parent compound), its semi-synthetic derivatives (**2**–**39**), and carvacrol against eight strains of *H. pylori*. Antibiotic susceptibility according to EUCAST guidelines (Clinical Breakpoint Tables v. 11.0, valid from 1 January 2021) is reported for each *H. pylori* strain.

Compound	MIC/MBC (μg/mL)							
	190	23	110R	NCTC 11637	F1	F40/499	F4	F34/497
**1** (thymol)	128/128	128/128	128/128	128/128	64/64	64/64	64/64	64/64
**2**	˃128/˃128	128/˃128	˃128/˃128	˃128/˃128	128/128	128/128	˃128/˃128	˃128/˃128
**3**	64/64	64/128	64/64	128/128	32/32	32/32	64/128	128/128
**4**	128/128	128/128	128/128	64/128	128/128	64/64	64/64	64/64
**5**	˃128/˃128	˃128/˃128	˃128/˃128	˃128/˃128	128/128	˃128/˃128	˃128/˃128	128/128
**6**	128/128	128/128	128/128	128/128	128/128	128/128	128/128	64/64
**7**	128/128	128/128	128/128	128/128	128/128	64/64	128/128	128/128
**8**	64/128	64/128	128/128	64/64	128/128	128/128	128/128	128/128
**9**	64/64	128/128	32/32	128/128	16/16	16/16	64/128	128/128
**10**	64/128	64/128	128/128	128/128	64/128	64/64	64/128	64/64
**11**	64/64	64/128	128/128	64/128	64/64	64/64	64/64	64/64
**12**	32/32	64/128	128/128	128/128	16/64	16/16	64/128	64/128
**13**	128/128	64/128	64/128	128/128	128/128	64/128	128/128	64/64
**14**	128/128	128/128	64/128	64/64	128/128	32/32	128/128	64/64
**15**	32/32	64/64	32/64	64/64	16/16	16/16	32/64	64/64
**16**	64/128	64/64	64/128	64/64	32/32	32/32	128/128	64/128
**17**	32/32	64/128	64/64	64/128	16/32	8/16	64/64	32/64
**18**	64/128	128/128	128/128	128/128	32/32	32/32	32/32	64/128
**19**	64/128	64/128	128/128	64/64	32/64	32/32	32/32	128/128
**20**	128/˃128	˃128/˃128	˃128/˃128	˃128/˃128	64/64	64/64	˃128/˃128	128/128
**21**	128/˃128	˃128/˃128	˃128/˃128	˃128/˃128	64/128	64/128	˃128/˃128	128/128
**22**	128/˃128	˃128/˃128	128/˃128	128/˃128	32/64	32/64	˃128/˃128	64/64
**23**	128/˃128	128/˃128	128/˃128	˃128/˃128	˃128/˃128	˃128/˃128	˃128/˃128	˃128/˃128
**24**	16/16	16/16	16/16	16/16	16/16	4/4	32/32	32/32
**25**	32/32	32/64	64/64	64/64	16/32	8/16	32/32	32/64
**26**	64/64	64/128	˃128/˃128	64/128	16/64	8/16	32/32	128/128
**27**	64/128	32/32	64/64	32/32	32/32	16/16	32/32	64/128
**28**	64/128	128/128	128/128	128/128	32/32	16/16	32/32	128/128
**29**	64/128	32/64	64/128	64/64	16/32	16/32	128/128	32/32
**30**	128/128	64/128	128/128	128/128	8/8	16/16	32/32	64/64
**31**	64/64	128/128	128/128	128/128	16/64	16/16	32/64	64/128
**32**	16/32	64/128	64/128	64/128	16/16	16/64	8/16	32/32
**33**	128/128	64/128	128/128	128/128	32/32	32/32	32/32	128/128
**34**	64/64	128/128	64/64	128/128	8/16	4/8	64/64	64/64
**35**	32/32	32/32	32/64	32/32	16/32	4/4	64/128	16/32
**36**	32/64	32/32	16/16	32/32	32/32	32/32	32/32	128/128
**37**	˃128/˃128	128/˃128	˃128/˃128	˃128/˃128	128/˃128	˃128/˃128	˃128/˃128	128/˃128
**38**	32/64	32/64	32/64	32/32	8/8	4/4	8/16	16/32
**39**	16/16	32/64	32/64	16/32	16/32	16/32	32/64	16/32
carvacrol	64/64	64/64	64/64	64/64	64/64	32/64	64/64	16/32
Antibiotic susceptibility	MTZ*−* CLR*−* AMX*−*	MTZ*−* CLR*−* AMX*−*	MTZ+ CLR*−* AMX*−*	MTZ+ CLR*−* AMX*−*	MTZ*−* CLR+ AMX*−*	MTZ+ CLR+ AMX*−*	MTZ+ CLR+ AMX*−*	MTZ+ CLR+ AMX*−*

Antibiotic susceptibility: MTZ+ = metronidazole resistant (MIC > 8 μg/mL); MTZ- = metronidazole susceptible (MIC ≤ 8 μg/mL); CLR+ = clarithromycin resistant (MIC > 0.5 μg/mL); CLR- = clarithromycin susceptible (MIC ≤ 0.25 μg/mL); AMX+ = amoxicillin resistant (MIC > 0.125 μg/mL); AMX- = amoxicillin susceptible (MIC ≤ 0.125 μg/mL). Compounds selected for *in vitro* anti-proliferative activity assays against AGS cells are highlighted in grey.

**Table 2:** IC_50_ values of the selected and reference compounds are expressed as mean ± standard deviation (SD) of three experiments with quintuplicate determinations.

Compound	IC_50_ (μM) ^a^
**thymol (1)**	200 ± 6.5
**9**	100 ± 5.8
**15**	93.5 ± 7.6
**24**	365 ± 8.5
**25**	607 ± 9.5
**26**	396 ± 8.5
**30**	613 ± 9.9
**34**	600 ± 9.0
**35**	na
**36**	574 ± 9.0
**38**	194 ± 6.0
**39**	na
**Carvacrol ^b^**	300 ± 6.4
**5-FU**	82.3 ± 5.6

^a^ Data are expressed as mean ± SD, n = 3; na: not active at the maximum concentration tested (800 μM). ^b^ from [15].

**Table 3:** In silico evaluated physicochemical properties of the top-rated compounds **9**, **15**, and **38**.

Compound	9	15	38
**Molecular weight (MW) ^b^**	206.32	240.34	316.44
**H-bond acceptors (HBA)**	1	1	1
**H-bond donators (HBD)**	0	0	0
Consensus Log P_O/W_ *	4.22	4.52	5.78
**Lipinski violations**	0	1	1
**GI absorption**	high	high	low
**P-gp substrate**	no	no	no
**PAINS alerts**	no alert	no alert	no alert
**WLOGP ^a^**	4.30	4.55	6.21
**TPSA (Å^2^) ^a,b^**	9.23	9.23	9.23
**XLOGP3 ^b^**	5.00	5.25	6.50
**Log S** **(ESOL) ^b^**	−4.24	−4.87	−6.12
**Fraction Csp3 ^b^**	0.57	0.29	0.22
**N° of rotatable bonds ^b^**	5	4	5
**MLOGP**	3.87	4.36	5.48
**boiled-egg graph**	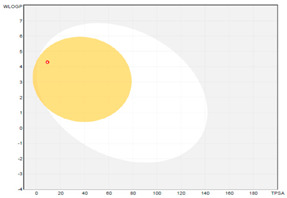	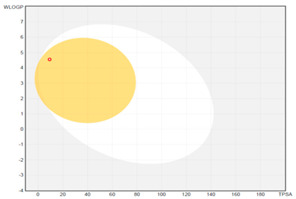	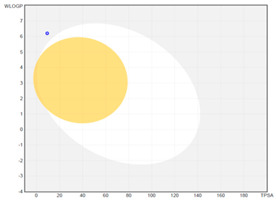
**bioavailability radar**	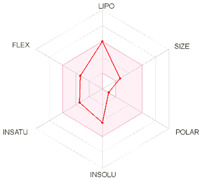	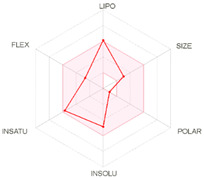	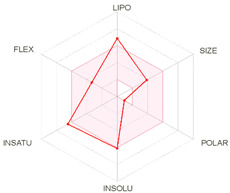

*Arithmetic mean of the values predicted by five in silico methods: XLOGP3, WLOGP, MLOGP, SILICOS-IT, iLOGP. Parameters range required to satisfy the Lipinski’s rule of five: MW < 500 g/mol, HBD < 5, HBA < 10, log P < 5. ^a^ Parameters used for the boiled-egg graph. ^b^ Parameters used for the bioavailability radar. Bioavailability radar parameters functional ranges: XLOGP3 between −0.7 and +5.0, MW between 150–500 g/mol, TPSA between 20–130 Å^2^, log S not higher than 6, saturation: fraction of carbons in the *sp^3^* hybridization not less than 0.25, and flexibility: no more than 9 rotatable bonds. Boiled-egg graph: white region: high probability of passive absorption by the gastrointestinal tract; yellow region: high probability of brain penetration.

## Supplementary Materials

The following are available online. Figure S1: NMR ^19^F spectrum at 564.7 MHz of **20** in CDCl_3_. Figure S2: NMR ^19^F spectrum at 564.7 MHz of **21** in CDCl_3_. Figure S3: NMR ^19^F spectrum at 564.7 MHz of **22** in CDCl_3_. Figure S4: NMR ^19^F spectrum at 564.7 MHz of **23** in CDCl_3_. Figure S5: NMR ^19^F spectrum at 564.7 MHz of **25** in CDCl_3_. Figure S6: NMR ^19^F spectrum at 564.7 MHz of **26** in CDCl_3_. Figure S7: NMR ^19^F spectrum at 564.7 MHz of **27** in CDCl_3_.

## Sample Availability

Samples of the compounds are available from the authors on request.
