# Supplementary material for: Synthesis and Evaluation of Thymol-Based Synthetic Derivatives as Dual-Action Inhibitors against Different Strains of H. pylori and AGS Cell Line
Source: Molecules. 2021 Mar 24;26(7):1829. doi: 10.3390/molecules26071829 (PMC8037164; doi:10.3390/molecules26071829)
Supplement: Supplementary file 1 [file molecules-26-01829-s001.pdf]

Synthesis and evaluation of thymol-based synthetic derivatives as dual-action inhibitors against different strains of *H. pylori* and AGS cell line

Francesca Sisto[1], Simone Carradori[2,*], Paolo Guglielmi[3], Mattia Spano[3], Daniela Secci[3], Arianna Granese[3], Anatoly P. Sobolev[4], Rossella Grande[2], Cristina Campestre[2], Maria Carmela Di Marcantonio[5], Gabriella Mincione[5]

1 Department of Biomedical, Surgical and Dental Sciences, University of Milan, 20122 Milan, Italy; francesca.sisto@unimi.it (F.S.)
2 Department of Pharmacy, "G. d'Annunzio" University of Chieti-Pescara, Via dei Vestini 31, 66100 Chieti, Italy; rossella.grande@unich.it (R.G.); cristina.campestre@unich.it (C.C.)
3 Department of Chemistry and Technology of Drugs, Sapienza University of Rome, P.le A. Moro 5, 00185 Rome, Italy; paolo.guglielmi@uniroma1.it (P.G.); mattia.spano@uniroma1.it (M.S.); daniela.secci@uniroma1.it (D.S.); arianna.granese@uniroma1.it (A.G.)
4 Institute for Biological Systems, "Annalaura Segre" Magnetic Resonance Laboratory, CNR, 00015 Monterotondo (Rome), Italy; anatoly.sobolev@cnr.it (A.P.S.)
5 Department of Innovative Technologies in Medicine and Dentistry, "G. d'Annunzio" University of Chieti-Pescara, Chieti, Italy; dimarcantonio@unich.it (M.C.D.M.); gabriella.mincione@unich.it (G.M.)

* Correspondence: Simone Carradori Ph.D. simone.carradori@unich.it (S.C.)

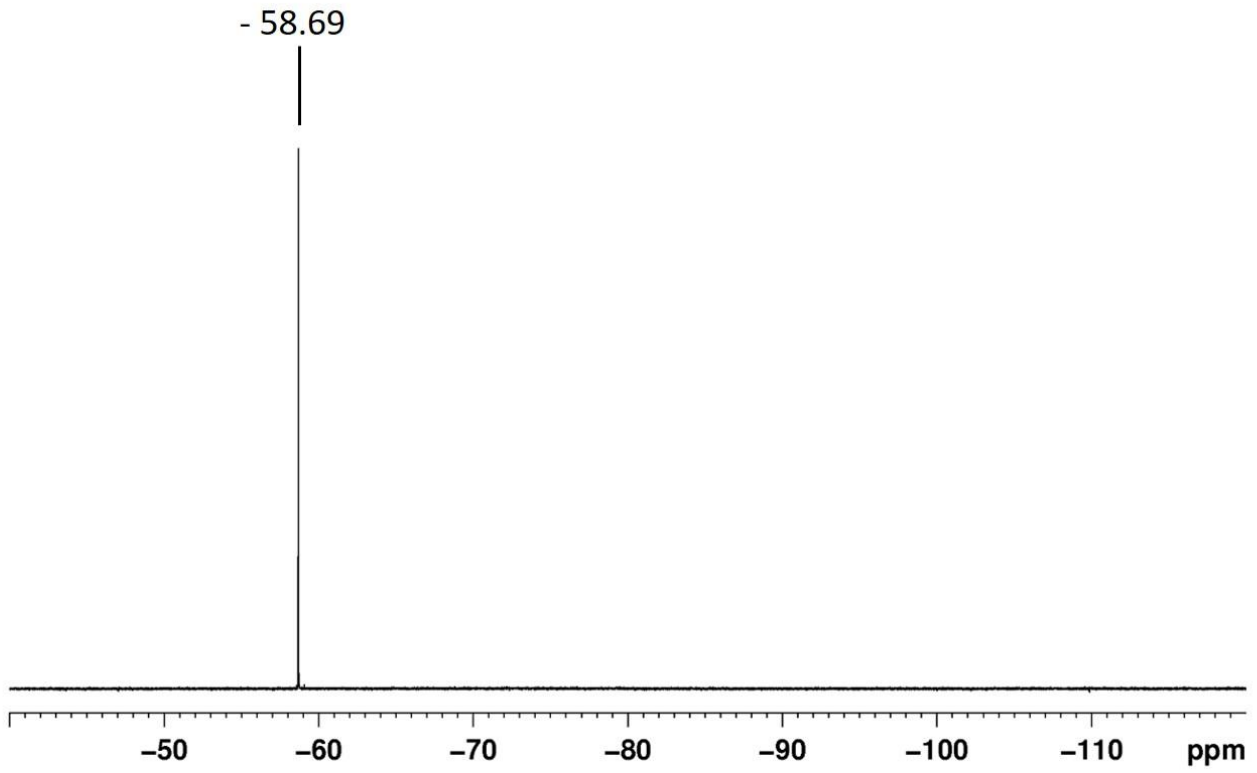

Figure S1. NMR ^{19}F spectrum at 564.7 MHz of **20** in $CDCl_3$.

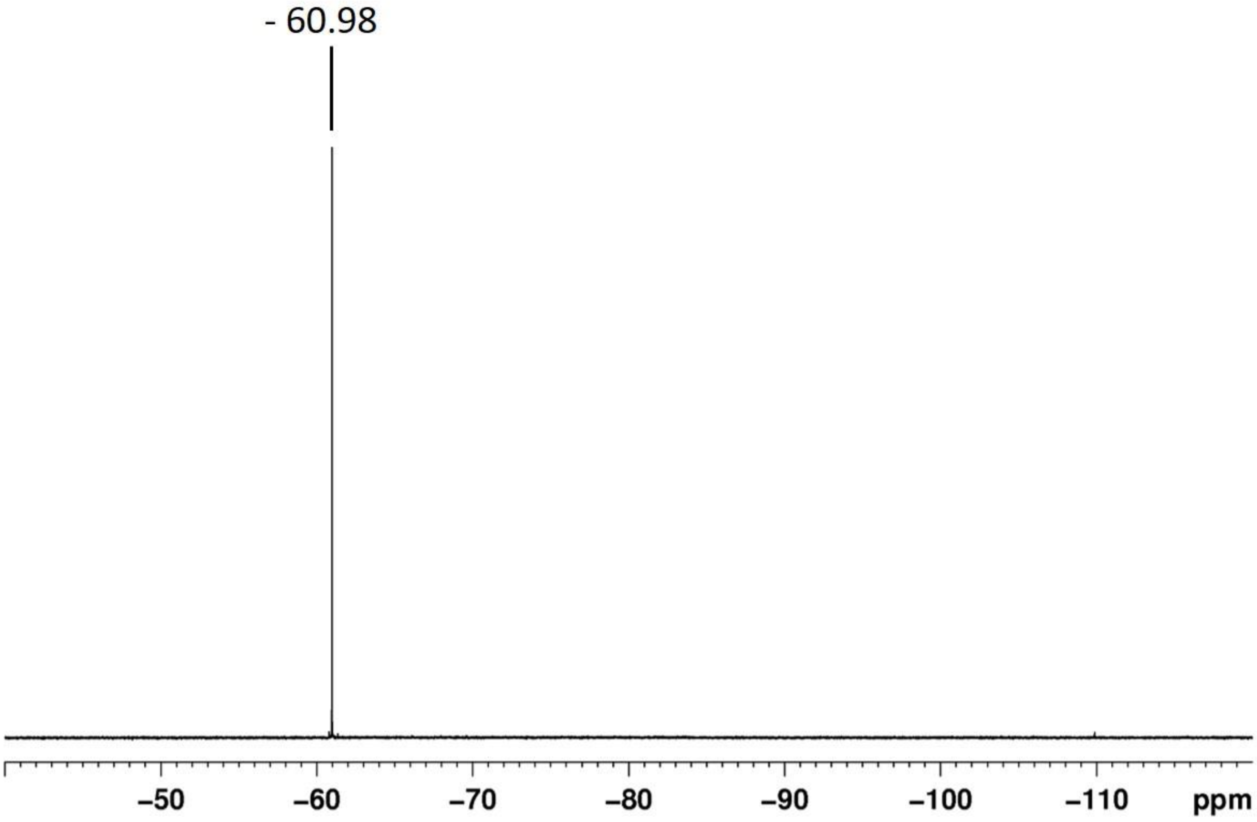

Figure S2. NMR ^{19}F spectrum at 564.7 MHz of **21** in $CDCl_3$.

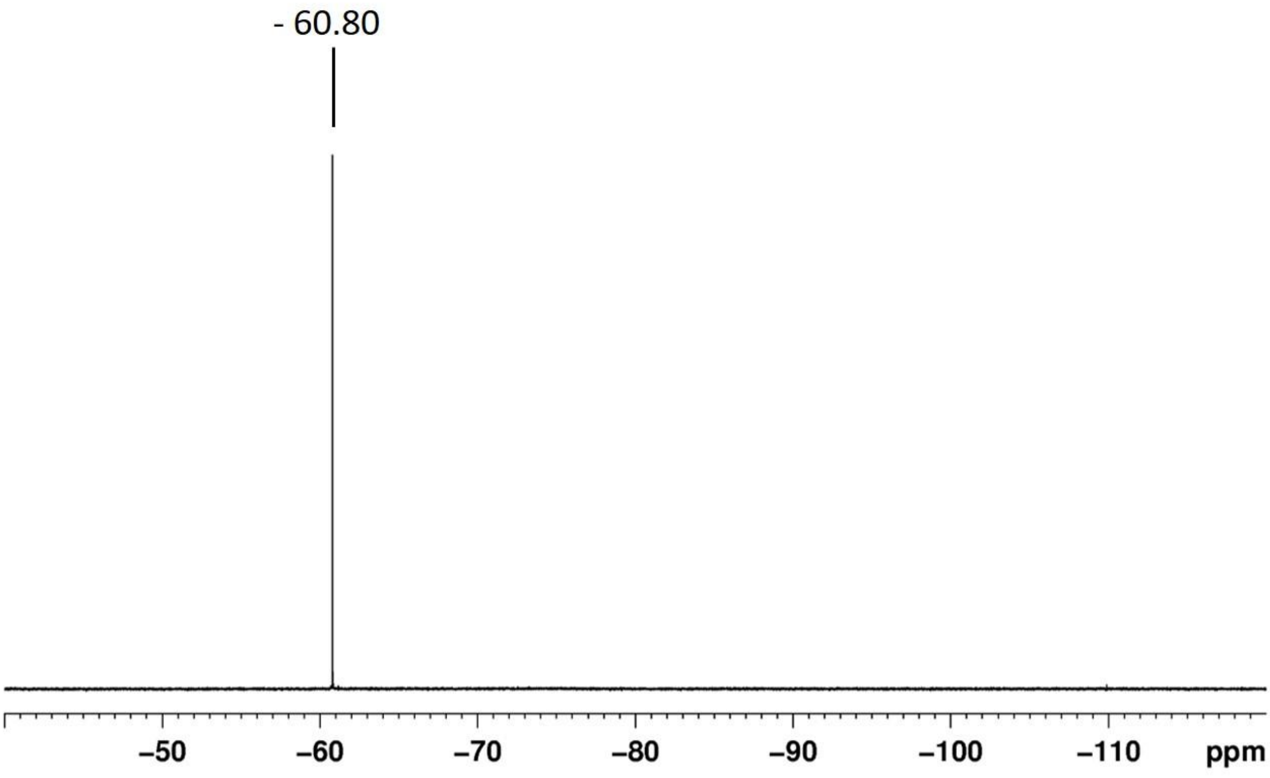

Figure S3. NMR ^{19}F spectrum at 564.7 MHz of **22** in $CDCl_3$.

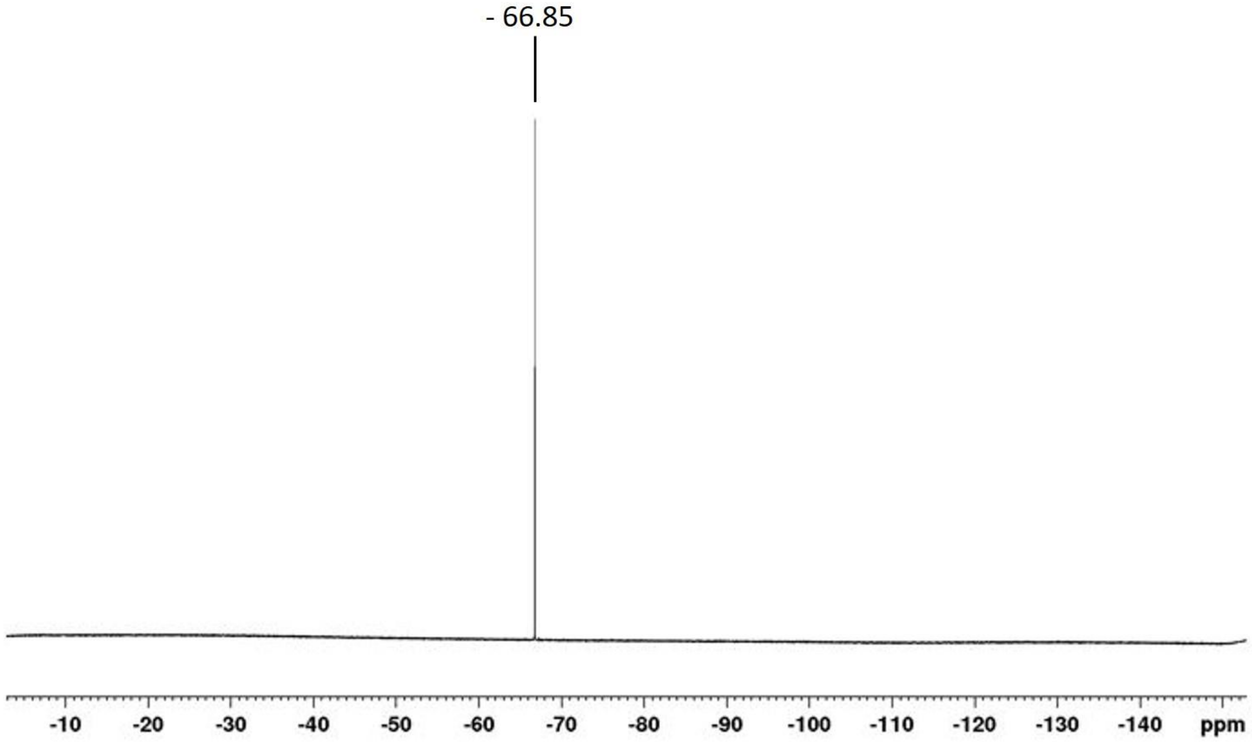

Figure S4. NMR ^{19}F spectrum at 564.7 MHz of **23** in $CDCl_3$.

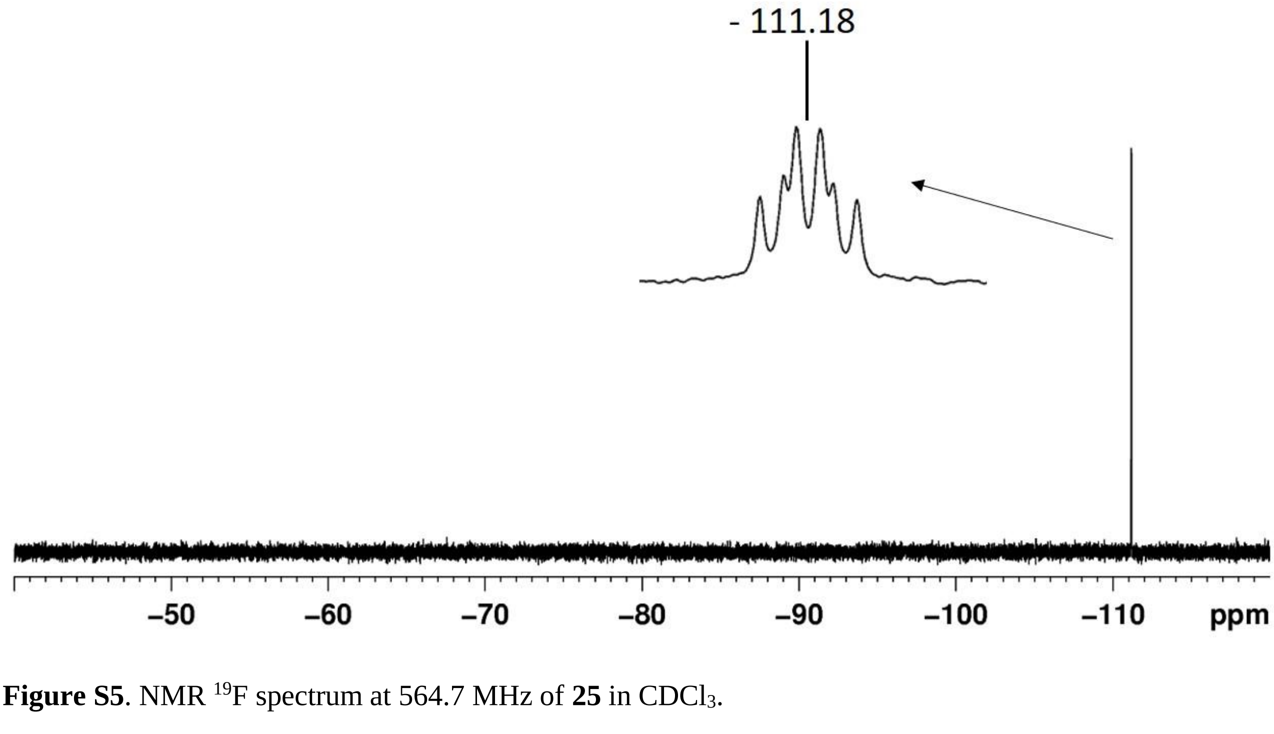

Figure S5. NMR ^{19}F spectrum at 564.7 MHz of **25** in $CDCl_3$.

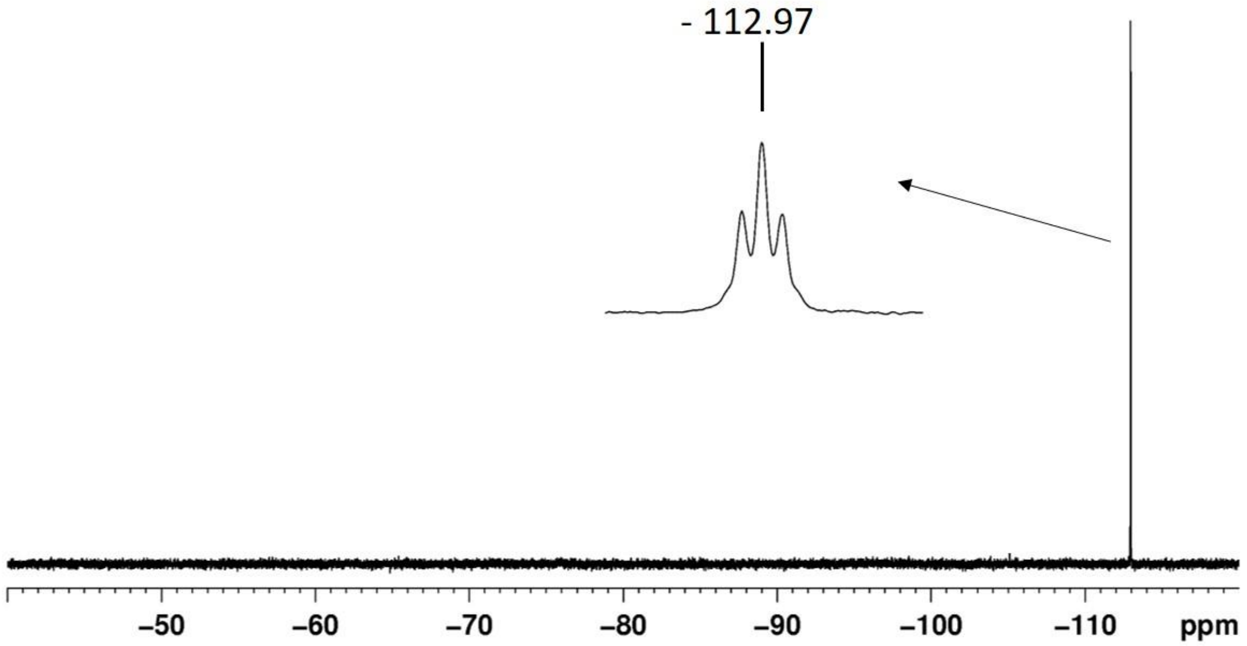

Figure S6. NMR ^{19}F spectrum at 564.7 MHz of **26** in $CDCl_3$.

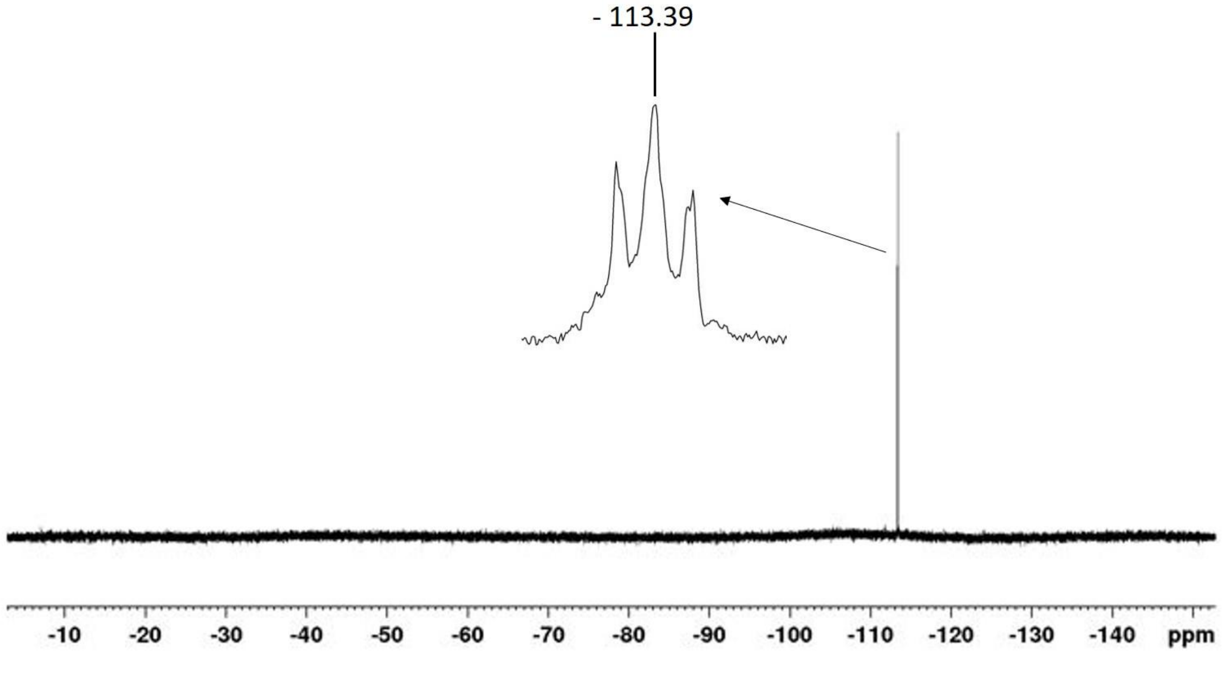

Figure S7. NMR ^{19}F spectrum at 564.7 MHz of **27** in $CDCl_3$.
